# Targeting FoxO proteins induces lytic reactivation of KSHV for treating herpesviral primary effusion lymphoma

**DOI:** 10.1371/journal.ppat.1011581

**Published:** 2023-08-18

**Authors:** Jungang Lan, Yeqing Wang, Shusheng Yue, Duo Xu, Yinan Li, Xiangyu Peng, Jiao Hu, Enguo Ju, Shanping He, Tingting Li

**Affiliations:** 1 State Key Laboratory of Developmental Biology of Freshwater Fish, Hunan International Joint Laboratory of Animal Intestinal Ecology and Health, Laboratory of Animal Nutrition and Human Health, Hunan Provincial Key Laboratory of Animal Intestinal Function and Regulation, College of Life Sciences, Hunan Normal University, Changsha, Hunan, China; 2 Center for Nanomedicine, The Third Affiliated Hospital, Sun Yat-sen University, Guangzhou, China; Harvard University, UNITED STATES

## Abstract

Kaposi’s sarcoma-associated herpesvirus (KSHV) is an oncogenic virus consisting of both latent and lytic life cycles. Primary effusion lymphoma (PEL) is an aggressive B-cell lineage lymphoma, dominantly latently infected by KSHV. The latent infection of KSHV is persistent and poses an obstacle to killing tumor cells. Like the "shock and kill" strategy designed to eliminate latent HIV reservoir, methods that induce viral lytic reactivation in tumor latently infected by viruses represent a unique antineoplastic strategy, as it could potentially increase the specificity of cytotoxicity in cancer. Inspired by this conception, we proposed that the induction of KSHV lytic reactivation from latency could be a potential therapeutic stratagem for KSHV-associated cancers. Oxidative stress, the clinical hallmark of PEL, is one of the most prominent inducers for KSHV reactivation. Paradoxically, we found that hydrogen peroxide (H_2_O_2_) triggers robust cytotoxic effects on KSHV-negative rather than KSHV-positive B lymphoma cells in a dose-dependent manner. Mechanistically, we identified forkhead box protein O1 (FoxO1) and FoxO3 as irrevocable antioxidant defense genes and both of them are upregulated by KSHV latent infection, which is essential for the promoted ROS scavenging in KSHV-positive B lymphoma cells. Pharmacological inhibition or functional knockdown of either FoxO1 or FoxO3 is sufficient to ablate the antioxidant ability and therefore increases the intracellular ROS level that further reverses KSHV from latency to active lytic replication in PEL cells, resulting in tremendous cell death both *in vitro* and *in vivo*. Additionally, the elevated level of ROS by inhibiting FoxO proteins further sensitizes PEL cells to ROS-induced apoptosis. Our study therefore demonstrated that the lytic reactivation of KSHV by inhibiting FoxO proteins is a promising therapeutic approach for PEL, which could be further extended to other virus-associated diseases.

## Author summary

Primary effusion lymphoma (PEL) is an aggressive malignancy caused by Kaposi’s sarcoma-associated herpesvirus (KSHV) latent infection. There are currently limited options for curing patients with PEL. Inspired by the "shock and kill" strategy used to eliminate latent HIV reservoir, we proposed that the induction of KSHV lytic reactivation would be a specific therapeutic regimen for PEL. In this study, we found that KSHV latent infection of B lymphoma cells significantly enhance the antioxidant capacity by upregulating both forkhead box protein O1 (FoxO1) and FoxO3. Inhibition of either FoxO1 or FoxO3 is sufficient to increase the intracellular ROS level that further induces KSHV lytic reactivation and subsequently causes massive cell death of PEL both *in vitro* and *in vivo*, which provides new opportunities for PEL treatment.

## Introduction

Kaposi’s sarcoma-associated herpesvirus (KSHV) is an oncogenic γ-herpesvirus that is etiologically related to Kaposi’s sarcoma (KS), primary effusion lymphoma (PEL), KSHV inflammatory cytokine syndrome (KICS) and a subset of multicentric Castleman’s diseases (MCD) [1,2]. Similar to other herpesviruses, KSHV has two life cycles: the quiescent latency and replicative lytic stages [3]. During viral lytic replication, most viral genes, such as RTA, PAN RNA, ORFK8, ORF57, and ORF65, get expressed in a cascade fashion, leading to the generation of infectious virions [4,5]. The majority of KS and PEL tumor cells are latently infected by KSHV and therefore only express limited viral products, such as LANA, indicating the essential role of KSHV latent infection in the development of KS and PEL tumors [3,4].

PEL is an extremely aggressive B-cell malignancy, often occurring in immunodeficient patients, such as human immunodeficiency virus (HIV)-positive individuals, with an overall median survival around 5 months [6]. Although existing antiviral therapies, such as highly active antiretroviral therapy (HAART), have reduced the number of AIDS-related patients, their benefits for PEL are yet limited [7]. Therefore, more effective and rationally designed therapeutic approaches are urgently required for PEL treatment. Recently, a stratagem termed “shock and kill” is designed to eliminate HIV by a two-step process. Firstly, latency reversing agents are applied to reactivate latent HIV (shock). Then, the reactivated cells are killed by immune cells or anti-HIV drugs [8–10]. By applying the similar concept, the induction of viral lytic replication could be a unique and specific antineoplastic strategy for virus-associated tumors. Several antiviral drugs, such as Cambogin and PEP005, are reported to induce substantial host cell destruction *in vitro* by reactivating latent KSHV [11–13]. However, their effectiveness and specificity *in vivo* yet remain big concerns. Therefore, more effective and specific therapeutic candidates for KSHV-associated malignancies are urgently required.

Reactive oxygen species (ROS) is a natural metabolic byproduct and a double-edged sword [14,15]. While the low level of ROS is essential for cell growth, modest to high concentrations of ROS can be toxic, resulting in cell death [15]. The abnormal hyper-proliferation of cancer cells is always accompanied by high metabolic rates and therefore excessive production of ROS, namely oxidative stress [16]. To cope with that, tumor cells have developed sophisticated antioxidant systems to avoid ROS-induced apoptosis and senescence [6]. Multiple external and internal stimuli, such as inflammation and oxidative stress, proangiogenic cytokines, and hypoxia, are reported to provoke KSHV reactivation. Among these, inflammation and oxidative stress are characteristic hallmarks of KSHV-associated tumors clinically [17–19]. Others and our group have previously shown that oxidative stress, such as hydrogen peroxide (H_2_O_2_), dramatically induces KSHV lytic reactivation, leading to massive cell death [20–22]. Paradoxically, the majority of KSHV persists as *bona fide* latency in PEL patients even exposure to oxidative stress [23]. Thus, PEL cells are speculated to develop superior detoxification systems to antagonize oxidative stress. The disruption of those systems may induce cell death by reactivating latent KSHV, representing a promising therapeutic approach for PEL.

Several proteins, including superoxide dismutase 2 (SOD2), peroxisomes (catalase), glutathione peroxidase (GPx) and glutathione are shown to directly detoxify different species of ROS [24]. Forkhead box O proteins (FoxOs) are a class of transcription factors with four members in mammalians: FoxO1, FoxO3, FoxO4 and FoxO6, that directly transactivate SOD2 and catalase to efficiently defend ROS [25]. Conversely, overwhelming evidence indicates that oxidative stress activates FoxOs to further boost the antioxidant defense [26,27]. Hence, FoxOs are widely regarded as both sensors and inhibitors of oxidative stress [27,28]. Accordingly, we have previously shown that inhibition of FoxO1 alone is sufficient to induce ROS-dependent KSHV reactivation in iSLK-RGB-BAC16 cells [22]. All these suggest that FoxOs might be novel targets for KSHV-associated tumors by simultaneously participating in the regulation of intracellular ROS level and KSHV life cycle.

In this study, we evaluated the efficiency of KSHV lytic reactivation induced by inhibition of FoxO1 and FoxO3 in PEL cells. We explored the regulatory roles of FoxO1 and FoxO3 in KSHV life cycle and the survival of KSHV-positive B lymphoma cells both *in vitro* and *in vivo*. We found that KSHV latent infection slightly while robustly upregulates FoxO1 and FoxO3, respectively, which is essential for KSHV-positive B lymphoma cells to defend oxidative stress. Inhibition of either FoxO1 or FoxO3 is sufficient to attenuate the cellular antioxidant capacity and subsequently increases the intracellular ROS level, resulting in KSHV lytic replication and massive death in KSHV-positive rather than KSHV-negative B lymphoma cells. Notably, by inducing the oxidative stress, FoxO inhibitor prominently represses the initiation and progression of PEL in a xenograft mouse model. These results together corroborated the specificity and effectiveness of the "shock and kill" stratagem as a unique therapeutic approach for malignancies caused by persistent viral latent infection.

## Results

### KSHV latent infection enhances the antioxidant capacity of B lymphoma cells

**Fig 1:**
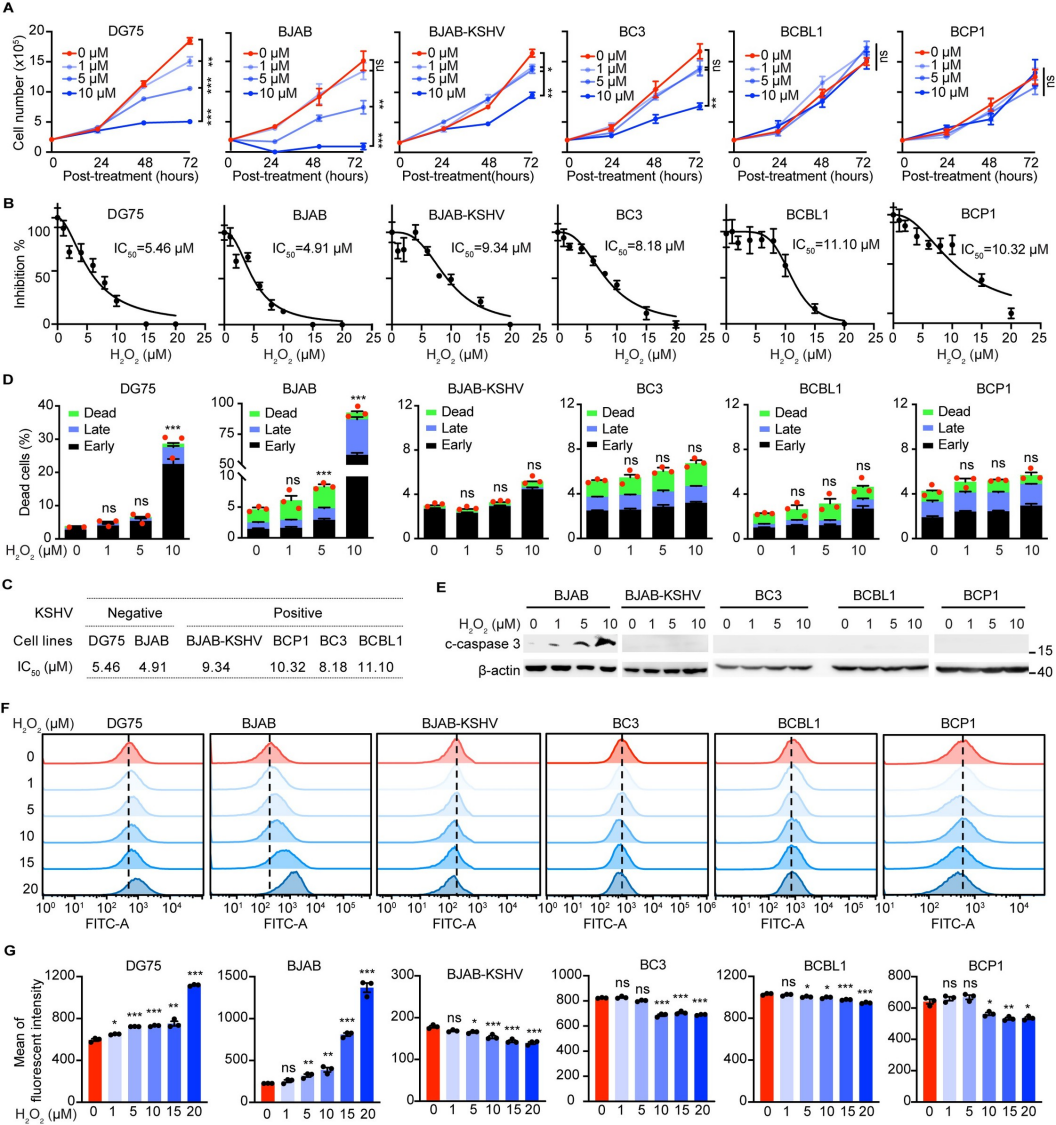
KSHV latent infection enhances the antioxidant capacity of B lymphoma cells. (A) The proliferation curves of DG75, BJAB, BJAB-KSHV and PEL (BC3, BCBL1 and BCP1) cells following H_2_O_2_ treatment for 24 h, 48 h, and 72 h. (B-C) The cell viability following H_2_O_2_ treatment for 72 h was indicated (B), and the concentration of H_2_O_2_-induced 50% cell death (IC_50_) was calculated from panel B (C). (D) Flow cytometry analysis of apoptosis using Annexin V and Propidium Iodide (PI) staining in DG75, BJAB, BJAB-KSHV and PEL cells after 72 h post-treatment of H_2_O_2_. Cells unstained with both Annexin V and PI indicated live cells; cells only stained with Annexin V indicated early apoptosis; cells stained with both Annexin V and PI indicated late apoptosis and cells only stained with PI indicated dead cells. (E) Western blotting analysis of c-caspase 3 protein expression after treating BJAB, BJAB-KSHV and PEL cells with H_2_O_2_ for 72 h. (F-G) Flow cytometry detection of the ROS levels using H2DCFDA staining in DG75, BJAB, BJAB-KSHV and PEL cells treated with H_2_O_2_ for 1 h. Representative images were shown in (F), and the quantification of F from three independent repeats were presented in (G). *, *P* < 0.05, **, *P* <0.01, ***, *P* < 0.001, ns, not significant compared to 0 μM H_2_O_2_.

H_2_O_2_, the most common molecule of ROS species in non-immune cells, is prominently produced as a byproduct of mitochondrial respiration [29,30]. It is previously reported that H_2_O_2_ at the high concentration of 500 μM induces KSHV lytic replication in PEL cells [20]. We then examined the physiological effects of H_2_O_2_ on the proliferation of three PEL cells (BCBL1, BC3 and BCP1). Because of no perfect controls for PEL cells, we included BJAB and DG75 that are KSHV-negative Burkitt’s lymphoma cells for references. Whereas H_2_O_2_ significantly inhibited the proliferation of KSHV-negative B lymphoma cells (BJAB and DG75) in a dose- and time-dependent manner, KSHV-positive B lymphoma cells including KSHV latently infected BJAB (BJAB-KSHV) and three PEL cells were paradoxically resistant to H_2_O_2_, as only the highest concentration of H_2_O_2_ at 10 μM showed marginally inhibitory effects on the proliferation of BC3, but not BCP1 and BCBL1 cells (Fig 1A). We further calculated the half maximal inhibitory concentration (IC_50_) of H_2_O_2_ for each cell line. Apparently, the IC_50_ of DG75 and BJAB (5.46 μM and 4.91 μM) was lower than that of BJAB-KSHV and PEL cells (9.34 μM, 8.18 μM, 11.10 μM and 10.32 μM for BJAB-KSHV, BC3, BCBL1 and BCP1, respectively) (Fig 1B and 1C). In agreement with that, H_2_O_2_ induced the apoptosis of KSHV-negative rather than KSHV-positive B lymphoma cells in a dose-dependent mode (Fig 1D). Consistently, the induction of caspase 3 cleavage (c-caspase 3) that is a characterized maker of apoptosis was only detected in BJAB cells, but not BJAB-KSHV and PEL cells (Fig 1E), indicating that KSHV-positive B lymphoma cells are more resistant to H_2_O_2_ than KSHV-negative B lymphoma cells. We further quantified the intracellular ROS level in these cells. Whereas H_2_O_2_ induced the accumulation of ROS in a dose-dependent manner in BJAB and DG75 cells, the ROS level was instead gradually decreased in BJAB-KSHV and PEL cells with the same treatment of H_2_O_2_ (Fig 1F and 1G). Taken together, these results indicated that KSHV latent infection enhanced the antioxidant defense system or genes in B lymphoma cells.

### FoxO proteins act as crucial antioxidant effectors in PEL cells

**Fig 2:**
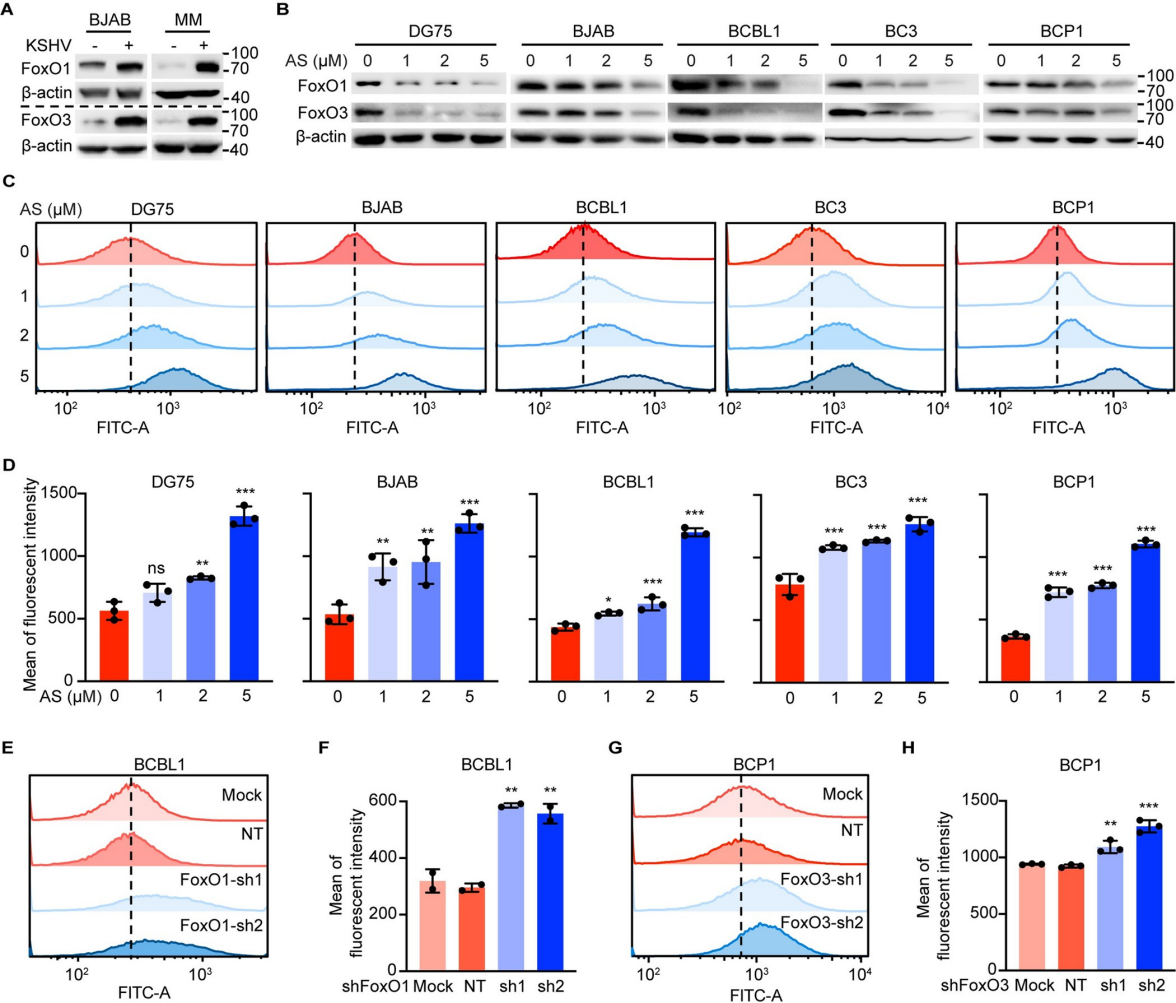
Inhibition of FoxO1 and FoxO3 increases the intracellular ROS level. (A) Western blotting analysis of FoxO1 and FoxO3 protein expression in BJAB and MM cells before and after KSHV latent infection. (B) Western blotting detection of FoxO1 and FoxO3 protein levels in BJAB, DG75 and PEL cells treated with AS1842856 at 0 (DMSO), 1, 2, or 5 μM for 48 h. (C-D) Flow cytometry detection of the intracellular ROS level using c-H2DCFDA staining in BJAB, DG75 and PEL cells treated with AS1842856 at 0 (DMSO), 1, 2, or 5 μM for 48 h. Representative images were shown in (C), and the quantification from 3 independent repeats were graphed in (D). (E-F) Flow cytometry detection of ROS using H2DCFDA staining in BCBL1 cells untransduced, transduced with non-targeting (NT) shRNA or FoxO1 shRNAs for 72 h. Representative images were shown in (E), and the quantification for E from three independent repeats, were presented in (F). (G-H) Flow cytometry detection of ROS using H2DCFDA staining in BCP1 cells untransduced, transduced with NT shRNA or FoxO3 shRNAs for 72 h. Representative images were shown in (G), and the quantitative results of three independent repeats, were presented in (H). AS in this figure represents AS1842856. *, *P* < 0.05, **, *P* <0.01, ***, *P* < 0.001, ns, not significant compared to 0 μM AS1842856 or NT.

We have recently reported that KSHV latent infection significantly upregulate FoxO1 and FoxO3 to ablate oxidative stress in KSHV-transformed cells [31]. Consistently, KSHV latent infection slightly upregulated FoxO1 while dramatically upregulated FoxO3 in BJAB cells (Fig 2A). Moreover, the protein expression of both FoxO1 and FoxO3 was robustly increased following KSHV latent infection of rat primary metanephric mesenchymal precursor (MM) cells (Fig 2A). To test whether KSHV upregulation of FoxO1 and FoxO3 accounted for the enhanced antioxidant capacity in KSHV-positive B lymphoma cells, we treated cells with AS1842856, a cell-permeable inhibitor that predominantly suppresses the transactivation activity of FoxO1 and FoxO3 [32]. Intriguingly, we found that AS1842856 efficiently reduced both FoxO1 and FoxO3 protein levels in DG75, BJAB and PEL cells in a dose-dependent manner (Fig 2B). We then examined the intracellular ROS level by a fluorescent sensor either in the presence or absence of AS1842856. Notably, AS1842856 consistently induced ROS accumulation in a dose-dependent manner in BJAB and three PEL cells (Fig 2C and 2D). Specifically, AS1842856 at 1, 2, and 5 μM increased the relative ROS intensity from 535 for DMSO-treated cells (0 μM) to 914, 953, and 1262 in BJAB cells, from 437 for 0 μM to 545, 623 and 1195 in BCBL1 cells, from 785 for 0 μM to 1080, 1132 and 1267 in BC3 cells, and from 366 for 0 μM to 719, 772 and 1262 in BCP1 cells (Fig 2D). In agreement with these results, knockdown of FoxO1 in BCBL1 cells that had the highest endogenous FoxO1 protein expression among three PEL cells increased the intracellular ROS level (Figs 2E, 2F, S1 and S2A). Similarly, silencing of FoxO3 in BCP1 cells that had a relatively high endogenous FoxO3 protein level was sufficient to increase the intensity of ROS level (Figs 2G, 2H, S1 and S2A). Of note, H_2_O_2_ alone at all indicated concentrations was unable to induce the ROS accumulation in BC3 cells (Figs 1F, 1G, S2B and S2C). In sharp contrast, when FoxO proteins were inhibited by AS1842856, the ROS level in BC3 cells was dramatically increased by H_2_O_2_ in a dose-dependent manner (S2B and S2C Fig), indicating the critical role of FoxO proteins in scavenging ROS. Altogether, these results suggested that KSHV hijacked both FoxO1 and FoxO3 to antagonize oxidative stress in B lymphoma cells.

### FoxO inhibitor AS1842856 suppresses the proliferation and induces the apoptosis of KSHV-positive B lymphoma cells

**Fig 3:**
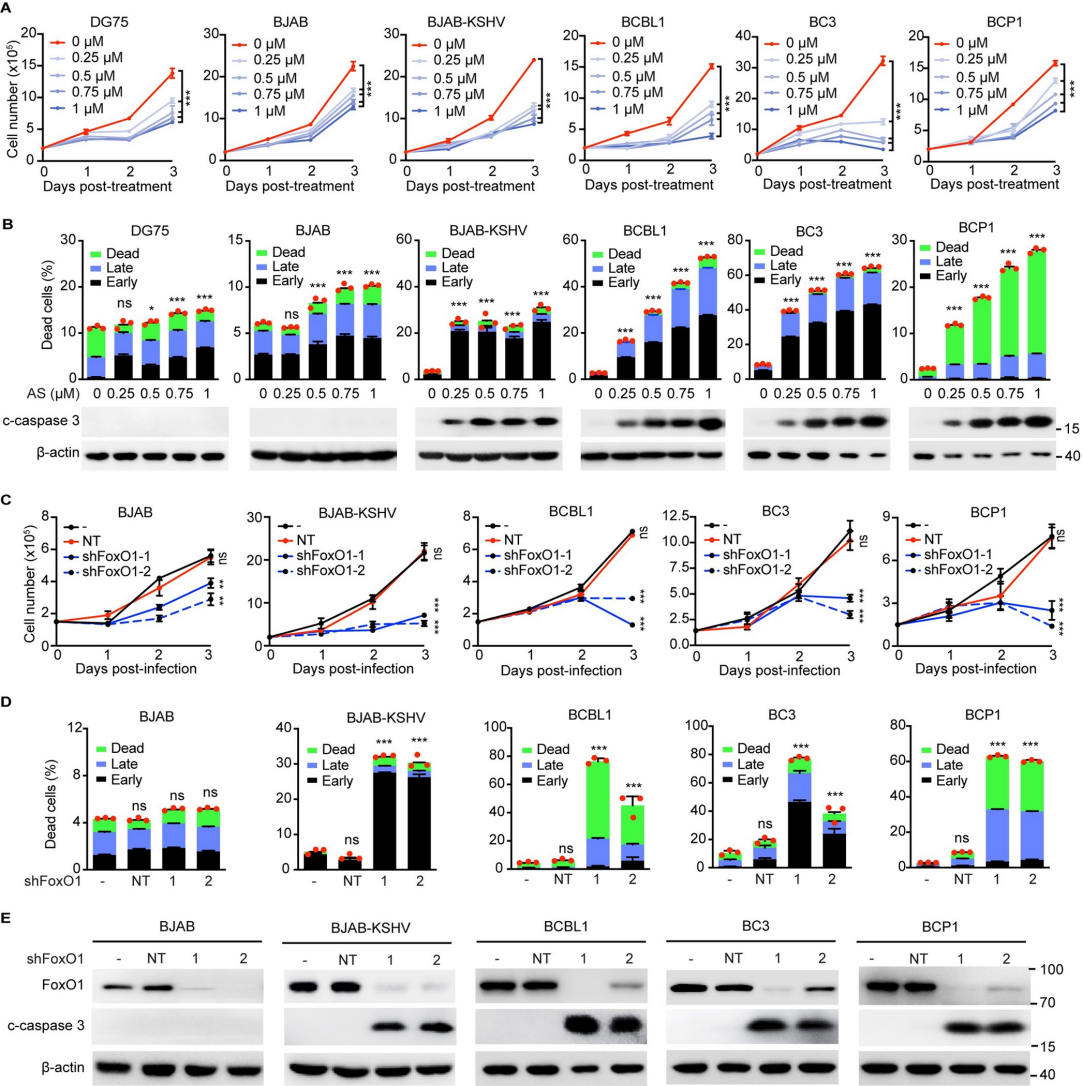
Inhibition of FoxO1 suppresses the proliferation and induces apoptosis of PEL cells. (A) The proliferation curves of DG75, BJAB, BJAB-KSHV and PEL cells following indicated concentrations of AS1842856 treatment for 24 h, 48 h, and 72 h. (B) Flow cytometry detection of apoptotic cells using Annexin V and PI staining in or Western blotting detection of c-caspase 3 protein level in DG75, BJAB, BJAB-KSHV and PEL cells treated with AS1842856 for 72 h. (C) The proliferation curves of BJAB, BJAB-KSHV and PEL cells untransduced, transduced with NT shRNA or FoxO1 shRNAs (1 or 2) for 1 day, 2 days, and 3 days. (D) Flow cytometry detection of apoptotic cells using Annexin V and PI staining in BJAB, BJAB-KSHV and PEL cells untransduced, transduced with NT, or FoxO1 shRNAs (1 or 2) for 72 h. (E) Western blot examination of FoxO1 and c-caspase 3 protein levels in BJAB, BJAB-KSHV and PEL cells untransduced, transduced with NT, or FoxO1 shRNAs (1 or 2) for 72 h. All the values shown were mean ± SEM from three independent experiments. AS in this figure represents AS1842856. **, *P* <0.05, **, *P* <0.01, ***, *P* < 0.001, ns, not significant compared to 0 μM AS1842856 or NT.

ROS accumulation is known to be detrimental for both normal and cancer cells. We thus examined the functional consequences of FoxOs regulation of ROS in PEL cell proliferation. As expected, AS1842856 significantly decreased the proliferation of BJAB-KSHV and PEL cells in a dose- and time-dependent manner (Fig 3A). AS1842856 also reduced the proliferation of DG75 and BJAB cells, but the inhibitory effect was much weaker than that seen in BJAB-KSHV and PEL cells (Fig 3A). Consistently, the IC_50_ of AS1842856 for BJAB was 0.60 μM that was much higher than that of BJAB-KSHV cells (0.23 μM) (S2D Fig). Furthermore, AS1842856 as low as 0.25 μM was sufficient to induce the apoptosis of BJAB-KSHV and PEL cells, which was further enhanced by the elevated concentrations of AS1842856 (Fig 3B). In contrast, the induction of apoptosis in DG75 and BJAB cells was much weaker than that seen in KSHV-positive B lymphoma cells (Fig 3B). The induction of apoptosis by AS1842856 in BJAB-KSHV and PEL cells was further confirmed by the increased protein level of c-caspase 3, which was not seen in DG75 and BJAB cells (Fig 3B). Taken together, these results indicated that KSHV-positive B lymphoma cells were more addicted to FoxO proteins and inhibition of FoxO proteins by AS1842856 preferentially suppressed the proliferation and survival of KSHV-positive B lymphoma cells by activating the apoptotic pathway.

As FoxO1 and FoxO3 are key antioxidant effectors in PEL cells, inhibition of both would reduce the antioxidant capacity and therefore make cells sensitive to ROS. As expect, the suppressed proliferation of BC3 cells by H_2_O_2_ was significantly enhanced by AS1842856 (S2E Fig). Consistently, H_2_O_2_ alone failed to induce the apoptosis of BC3 cells (Figs 1D and S2F). In sharp contrast, BC3 cells were instead susceptible to H_2_O_2_-induced apoptosis when pretreated with AS1842856 (S2F Fig). Altogether, these results indicated that inhibition of FoxO1 and FoxO3 dampened the antioxidant ability of PEL cells and therefore sensitized PEL cells to H_2_O_2_ treatment.

### FoxO1 or FoxO3 knockdown inhibits the proliferation and induces apoptosis of PEL cells

As AS1842856 inhibits both FoxO1 and FoxO3, we dissected their roles by shRNA-mediated specific knockdown. All shRNAs targeting either FoxO1 or FoxO3 showed satisfactory knockdown efficiency (S1 and S3A Figs). Knockdown of either FoxO1 or FoxO3 preferentially inhibited the proliferation of BJAB-KSHV and PEL cells (Figs 3C and S3B). Specifically, the cell number of BJAB-KSHV and PEL cells including BCBL1, BC3 and BCP1 was decreased by 67.7%-76.4%, 57.2%-87.2%, 54.5%-70.1%, and 67.1%-81.6%, respectively, whereas only 29.1%-47.3% reduction of cell number was observed in BJAB, following lentiviral transduction of FoxO1 shRNAs for three days (Fig 3C). Likewise, the cell proliferation was reduced by 75.1%-76.6%, 61.6%-64.8%, 51.4%-54.2% and 49.0%-58.0% in BJAB-KSHV, BCP1, BC3, and BCBL1, while only minimal effect was observed in BJAB cells after transduction of FoxO3 shRNAs for three days (S3B Fig). In agreement with that, inhibition of either FoxO1 or FoxO3 by shRNAs triggered the apoptosis, accompanied by the increased expression of c-caspase 3 protein in BJAB-KSHV and PEL cells, but not in BJAB cells (Figs 3D, 3E and S3A and S3C). Additionally, simultaneous knockdown of both FoxO1 and FoxO3 could not additively or synergistically suppressed the proliferation and induced apoptosis of BC3 and BCBL1 cells (S3D–S3F Fig). These data suggested that that FoxO1 and FoxO3 were not functionally reductant, and both were indispensable for the proliferation and survival of KSHV-positive B lymphoma cells.

### Inhibition of FoxO proteins disrupts KSHV latency and induces viral lytic replication

**Fig 4:**
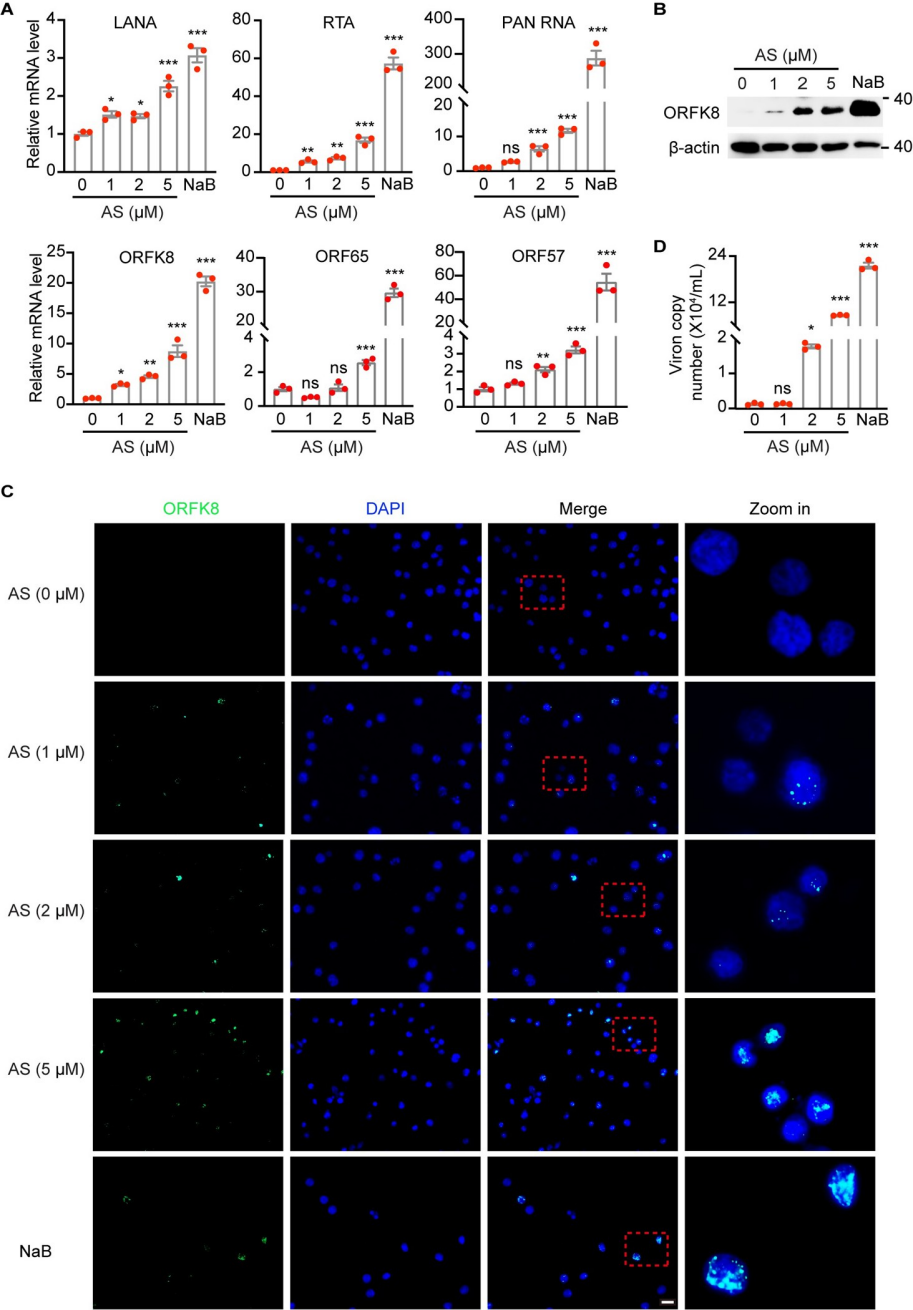
FoxO inhibitor AS1842856 induces KSHV lytic replication. (A) RT-qPCR analysis of the mRNA levels of KSHV LANA, RTA, PAN RNA, ORFK8, ORF65 and ORF57 in BCP1 cells treated with 0, 1, 2, 5 μM AS1842856 or 0.3 mM sodium butyrate (NaB) for 72 h. (B-C) The protein level of ORFK8 was examined by Western blot (B) and immunofluorescent assay (C), following 0, 1, 2, 5 μM AS1842856 or 0.3 mM sodium butyrate treatment for 72 h. Scale bar, 20 μm. (D) The quantitation of produced KSHV virion in the supernatant of BCP1 cells treated with 0, 1, 2, 5 μM AS1842856 or 0.3 mM NaB for 96 h by qPCR. AS in this figure represents AS1842856. *, *P* < 0.05, **, *P* <0.01, ***, *P* < 0.001, ns, not significant compared to 0 μM AS1842856.

ROS is reported to induce robust KSHV lytic reactivation from latency [21,33]. As we have proved that inhibition of FoxO proteins potently increased the intracellular ROS level (Fig 2C–2H), we then examined whether FoxO inhibition is sufficient to induce KSHV lytic replication in PEL cells. To test that, we firstly examined the transcriptional expression of viral lytic genes by RT-qPCR following AS1842856 treatment. We used sodium butyrate (NaB) as the positive control because of its potent induction of KSHV lytic reactivation [34]. AS1842856 dramatically and consistently increased the transcript levels of KSHV lytic genes, including RTA, PAN RNA, ORFK8, ORF65, and ORF57, in three PEL cells (Figs 4A and S4A and S4B). We also observed slight upregulation of latent LANA, which might account from RTA-mediated transcriptional activation (Figs 4A and S4A and S4B) [35]. The induction of ORFK8 protein by AS1842856 was also confirmed in BCP1 cells by both immunoblotting and immunofluorescent analyses (Fig 4B and 4C). Consistently, AS1842856 increased the production of KSHV progeny virions released on the supernatants in a dose-dependent manner (Figs 4D and S4C and S4D). Notably, the induction of KSHV lytic replication by AS1842856 at 5 μM in BCBL1 and BC3 cells was even comparable to NaB (S4A-S4D Fig).

**Fig 5:**
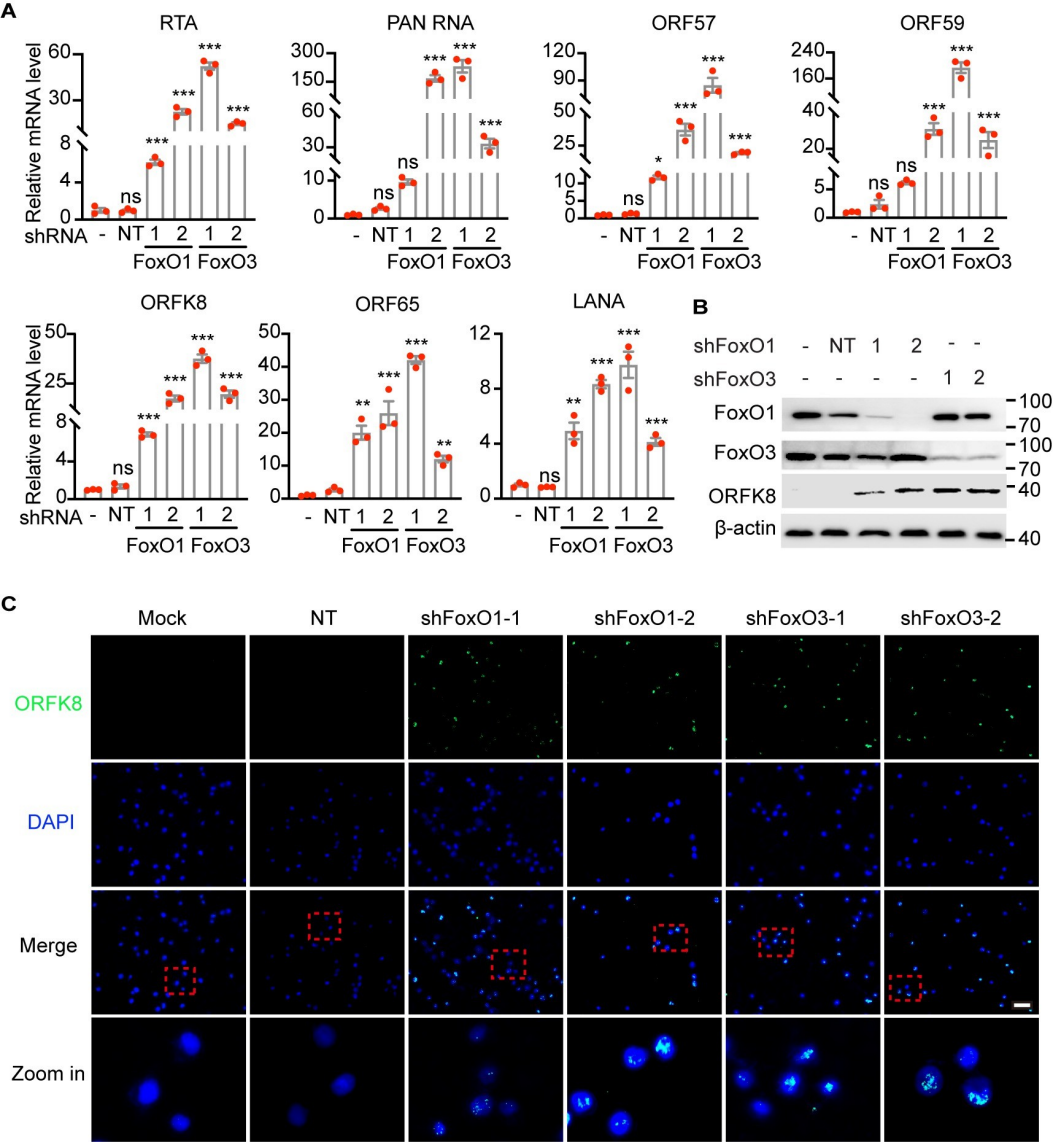
FoxO1 and FoxO3 knockdown triggers KSHV lytic replication. (A) The mRNA levels of KSHV RTA, PAN RNA, ORF57, ORF59, ORFK8, ORF65 and LANA in BCP1 cells was detected by RT-qPCR after 72 h post-transduction of NT shRNA, FoxO1 shRNAs (1 or 2), or FoxO3 shRNAs (1 or 2). (B-C) The protein expression of KSHV ORFK8 in BCP1 cells was examined by Western blot (B) and immunofluorescent assay (C) following the transduction of NT shRNA, FoxO1 shRNAs (1 or 2), or FoxO3 shRNAs (1 or 2) for 96 h. AS in this figure represents AS1842856. Scale bar, 20 μm. *, *P* < 0.05, **, *P* <0.01, ***, *P* < 0.001, ns, not significant by 2-tailed Student’s t test compared to NT.

To further verify the role of FoxOs in KSHV reactivation, we performed FoxO1 or FoxO3 knockdown by shRNAs. Consistent with AS1842856, shRNAs-mediated FoxO1 or FoxO3 knockdown robustly switched the viral life cycle from latency to lytic replication (Figs 5 and S5). Specifically, the transcription of KSHV lytic genes, including RTA, PAN RNA, ORFK8, ORF57 and ORF65, was dramatically increased in three PEL cells (Figs 5A and S5A and S5B). Further, the protein expression of ORFK8 was substantially increased in BCP1 cells following either FoxO1 or FoxO3 knockdown (Fig 5B and 5C). All together, these results demonstrated that FoxO proteins were key regulators of KSHV life cycle and inhibition of FoxO proteins induced robust lytic reactivation of KSHV.

### Inhibition of FoxO1 induces KSHV reactivation in a ROS-dependent manner

**Fig 6:**
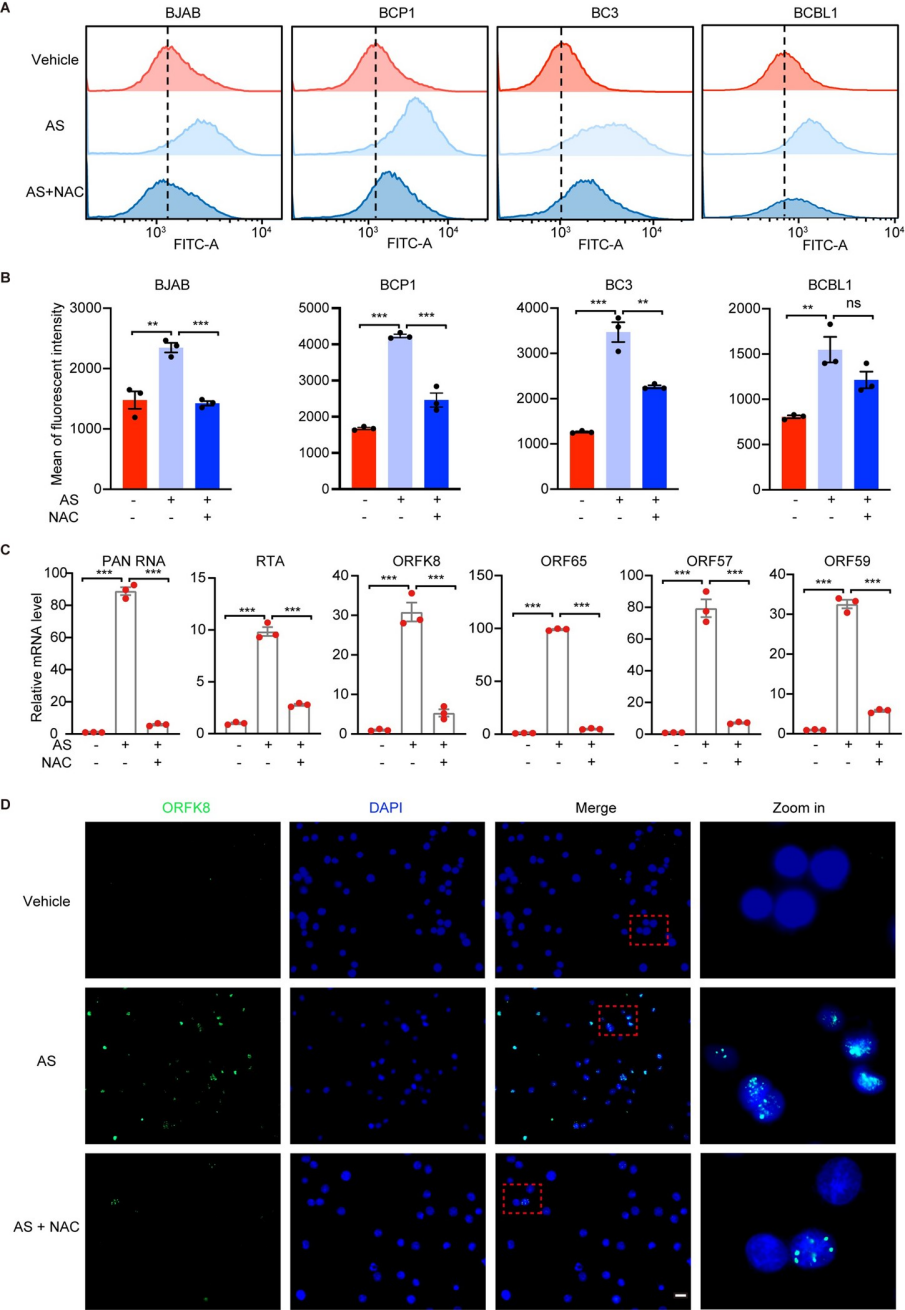
FoxO1 inhibition by AS1842856 triggers KSHV lytic reactivation in a ROS-dependent manner. (A-B) Flow cytometry detection of the intracellular ROS level using H2DCFDA staining in BJAB and PEL cells treated with vehicle or 5 μM AS1842856 with or without the daily treatment of 0.4 mM NAC for 3 days. Representative images were shown in (A), and the quantification from three independent repeats were presented in (B). (C) RT-qPCR detection of the transcript expression of viral lytic genes including PAN RNA, RTA, ORFK8, ORF65, ORF57, and ORF59 in BCP1 cells treated with 0 or 5 μM AS1842856 with or without the daily treatment of 0.4 mM NAC for 3 days. (D) The detection of ORFK8 protein expression by immunofluorescent assay in BCP1 cells treated with 0 or 5 μM AS1842856 with or without the daily treatment of 0.4 mM NAC daily for 3 days. AS in this figure represents AS1842856. Scale bar, 20 μm. *, *P* < 0.05, **, *P* <0.01, ***, *P* < 0.001, ns, not significant.

To further determine whether KSHV reactivation by FoxO1 inhibition is mediated by oxidative stress, we introduced N-acetyl cysteine (NAC), a potent ROS scavenger. As expected, FoxO inhibition by AS1842856 stably increased the intensity of ROS in BJAB and PEL cells, which was efficiently blocked by NAC (Fig 6A and 6B). In agreement with these results, NAC dramatically reduced the enhanced transcription of KSHV lytic genes, including PAN RNA, RTA, ORFK8, ORF57 and ORF59, following AS1842856 treatment (Figs 6C and S6A and S6B). Further, the induced expression of ORFK8 protein by AS1842856 was significantly reduced by NAC in BCP1 cells (Fig 6D).

**Fig 7:**
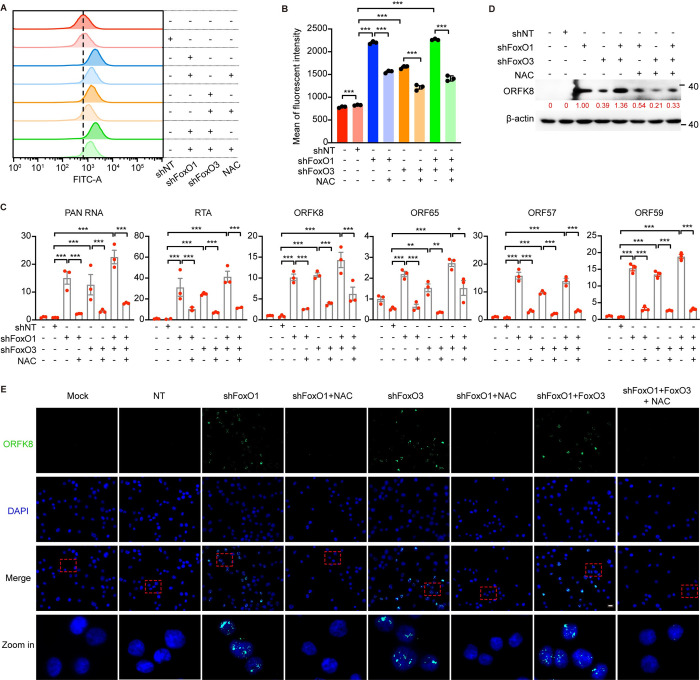
Knockdown of FoxO1, FoxO3, or both induces KSHV lytic reactivation in a ROS-dependent manner. (A-B) Flow cytometry detection of the intracellular ROS level using H2DCFDA staining in BCP1 cells untransduced, transduced with NT shRNA, FoxO1 shRNA-sh1, FoxO3 shRNA-sh1 or both, with or without the daily treatment of 0.4 mM NAC for 3 days. Representative images were shown in (A), and the quantification from three independent repeats were presented in (B). (C) RT-qPCR detection of the mRNA expression of KSHV lytic genes including PAN RNA, RTA, ORFK8, ORF65, ORF57 and ORF59 in BCP1 cells untransduced, transduced with NT shRNA, FoxO1 shRNA-sh1, FoxO3 shRNA-sh1 or both, with or without the daily treatment of 0.4 mM NAC for 3 days. (D-E) Western blotting (D) and immunofluorescent (E) detection of ORFK8 protein expression in BCP1 cells untransduced, transduced with NT shRNA, FoxO1 shRNA-sh1, FoxO3 shRNA-sh1 or both, with or without the daily treatment of 0.4 mM NAC for 3 days. Scale bar, 20 μm. *, *P* < 0.05, **, *P* <0.01, ***, *P* < 0.001 by 2-tailed Student’s t test compared to NT.

We further confirmed these results by treating FoxO-knockdown cells with NAC. Knockdown of FoxO1, FoxO3 or both increased the ROS levels in BCP1 cells, which was significantly blocked by NAC (Fig 7A and 7B). Consistently, the induction of the transcription of KSHV lytic genes, including PAN RNA, RTA, ORFK8, ORF65, ORF57, ORF59, following knocking down FoxO1, FoxO3 or both was robustly inhibited by NAC in BCP1 cells (Fig 7C). Further, knockdown of FoxO1, FoxO3 or both induced the expression of ORFK8 protein, which was efficiently prevented by NAC (Fig 7D and 7E). Taken together, these results indicated that KSHV lytic replication triggered by FoxO inhibition by either inhibitor or knockdown was highly dependent on ROS.

### FoxO inhibitor AS1842856 inhibits the initiation and progression of PEL *in vivo*

**Fig 8:**
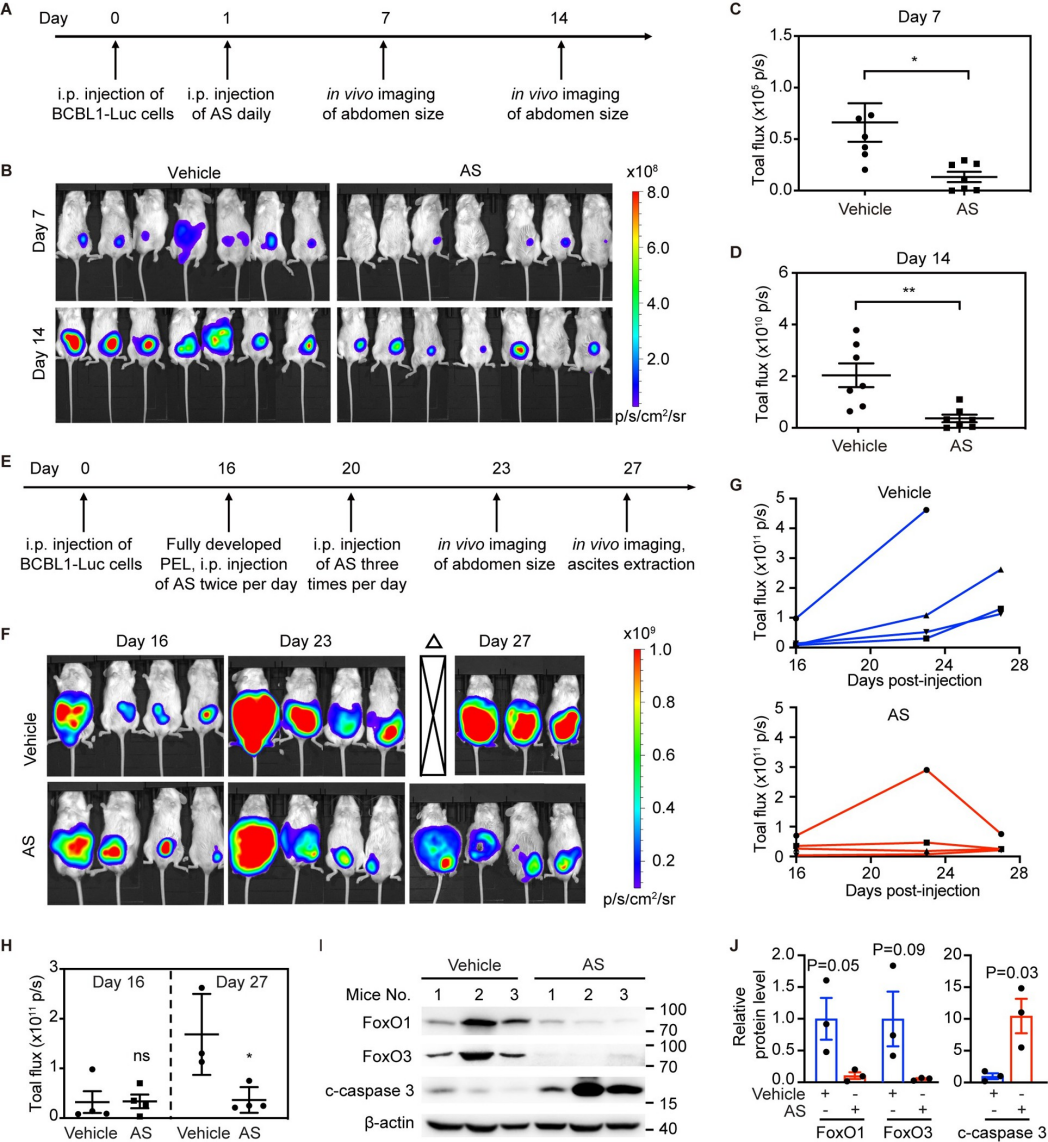
AS1842856 inhibits tumor initiation, progression and regression in a xenograft mouse model of PEL. (A) The experimental timeline for PEL initiation and progression. (B) Live imaging of mice treated with vehicle control or AS1842856 at day 7 and 14 post-inoculation of BCBL1-Luc cells. (C-D) The luminescence signals of PEL tumor in mice treated with AS1842856 at day 7 (C) and day 14 (D) post-inoculation of BCBL1-Luc cells. (E) The experimental timeline for PEL regression. (F) The luminescence images of mice before and after vehicle or AS1842856 treatments for 0, 7 and 11 days. The luminescence images of fully developed PEL treated with either vehicle or AS1842856 were shown at day 16, day 23 and day 27 post-inoculation of BCBL1-Luc cells, respectively. Open triangles indicated mice euthanized before the luminescence assay due to overwhelmed tumor burden. (G) The bioluminescence signals of PEL in individual mouse treated with vehicle or AS1842856 over time. (H) Inhibition of bioluminescence signals in mice treated with AS1842856 for 0 and 11 days. The total radiant efficiency of fully developed PEL treated with vehicle or AS1842856 were quantified at day 16 and day 27 post-inoculation of BCBL1-Luc cells, respectively. (I-J) Western blotting detection of FoxO1, FoxO3 and c-caspase 3 protein expressions in mice ascites treated with either vehicle or AS1842856. J was the quantification for panel I. *, *P* < 0.05, **, *P* <0.01.

The results so far indicated that FoxO1 and FoxO3 are the safeguards to sustain the proliferation and survival of PEL cells by defending oxidative stress and thereby holding KSHV in latency. We then tested the effects of KSHV reactivation by FoxO inhibition in an *in vivo* mouse model. To achieve that, we firstly constructed BCBL1 cells stably expressing a cassette of luciferase, nominated as BCBL1-Luc thereafter. We verified that the bioluminescence signal of BCBL1-Luc cells is linearly correlated with the cell number, which allowed us to monitor PEL growth *in vivo* (S7A and S7B Fig). We then created a xenograft mouse model by engrafting BCBL1-Luc cells into NOD-SCID mice. On the next day, the mice were intraperitoneally injected with AS1842856 or vehicle control twice per day (Fig 8A). No obvious changes were observed in organs including heart, liver, spleen, lung, and kidney following AS1842856 treatment (S7C Fig). Notably, seven out of seven mice (100%) in the control group in comparison with three out of seven mice (42.9%) in the treatment group developed PEL at day 7 post-inoculation (Fig 8B). Additionally, mice treated with AS1842856 had significantly lower intensity of bioluminescence signals than the control group, indicating an effective suppression of PEL initiation and progression by FoxO inhibitor *in vivo* (Fig 8C and 8D).

### FoxO inhibitor AS1842856 induces the regression of established PEL tumor

We further examined the therapeutic efficacy of FoxO inhibitor for grown PEL *in vivo* following the depicted timeline (Fig 8E). At day 16 post-inoculation of BCBL1-Luc cells, bioluminescence imaging was conducted to quantitate the tumor burden of individual mouse (Fig 8F and 8H). Based on the bioluminescence signals, mice were divided into two groups of which there were no statistically significant difference in the size of established PEL (Fig 8F and 8H). These two groups of mice were then treated with either vehicle or AS1842856. While PEL in the treated group has significant shrinkage as early as one-week treatment of AS1842856, PEL in the control group continued to grow, as indicated by the bioluminescence signals (Fig 8F and 8G). Furthermore, whereas the bioluminescence imaging detected strong signals in all mice treated with vehicle at day 11 post-treatment (day 27 post injection of BCBL1-Luc cells), mice treated with AS1842856 had much weaker signals at this time point (Fig 8F–8H). This suggested that FoxO inhibition potently induced the regression of grown PEL. We also observed solid tumors on the peritoneum and retroperitoneum in all vehicle-treated mice (S7D Fig) [36,37]. In sharp comparison, the solid tumors was only present in three out of four mice treated with AS1842856 (S7D Fig). Moreover, the size of solid tumors in the AS1842856-treated group was much smaller than that of control group (S7D Fig). Additionally, AS1842856 suppressed the PEL infiltration-induced splenic enlargement (S7E Fig).

To investigate the mechanism of AS1842856-induced PEL regression, we collected ascites from the control and treated mice and subsequently examined the expression of FoxO1 and FoxO3 protein. Consistent with results *in vitro*, both FoxO1 and FoxO3 protein levels were significantly decreased by AS1842856 *in vivo* (Fig 8I and 8J). Additionally, AS1842856 dramatically induced the expression of c-caspase 3 protein and the transcription of KSHV lytic genes including RTA, ORFK8, ORF59 and ORF65 (Figs 8I and 8J and S7F). Collectively, these results demonstrated that FoxOs suppression robustly induced the regression of established PEL by triggering KSHV lytic reactivation.

## Discussion

PEL is a KSHV-associated non-Hodgkin’s lymphoma that develops in the body cavity, commonly seen in immunodeficient patients, such as HIV-infected persons [38]. The prognosis of PEL patients is poor and no specific and efficient therapies are available so far [38,39]. The majority of PEL cells are latently infected by KSHV, which represents a trick for virus to quiescently and permanently persist in host cells [39,40]. Although only limited genes expressed during latency, these viral products endow both proliferative and anti-apoptotic signals to induce KSHV-associated malignancies [41–43]. Therefore, latency is widely considered as a curb to eliminate KSHV and its associated tumors [13,44].

The high level of ROS is the clinical feature of both PEL and KS tumors and is previously shown to effectively reactivate KSHV, leading to cell death [21,33]. In contrast, our results suggested that KSHV-positive cells are paradoxically more resistant to ROS than KSHV-negative cells, as H_2_O_2_ alone induced massive cell death in BJAB and DG75 cells, but not in KSHV-positive B lymphoma cells (Fig 1A and 1C). These results indicated the existence of enhanced antioxidant systems in PEL cells such that direct addition of ROS like H_2_O_2_ might not be an applicable remedy. Here we identified FoxO1 and FoxO3 as the key antioxidant genes and KSHV latent infection upregulated both of them, which helps PEL cells to resist oxidative stress (Figs 2 and S2B and S2C).

As the canonical downstream effector of the PI3K-AKT oncogenic pathway, FoxO proteins are widely considered as tumor suppressors by inhibiting survival-related pathways, such as transactivating apoptotic genes Bim and PUMA [45–47]. Conversely, compelling evidence corroborates the oncogenic functions of FoxOs as well [48,49]. For instance, FoxO proteins maintain the intracellular redox homeostasis, which ensures cell survival under stressful environments [48,50,51]. Additionally, FoxO1 and FoxO3 are frequently mutated or hyper-upregulated in leukemia, and pharmacological inhibition of FoxO1 dramatically reduces the aberrant growth of leukemia, indicating the fundamental roles of FoxO1 and FoxO3 in blood cancer [52,53]. Here, we characterized the essential role of FoxO1 and FoxO3 in maintaining KSHV latency and proposed that the induction of viral lytic replication by targeting FoxOs as a clinically beneficial remedy for PEL.

**Fig 9:**
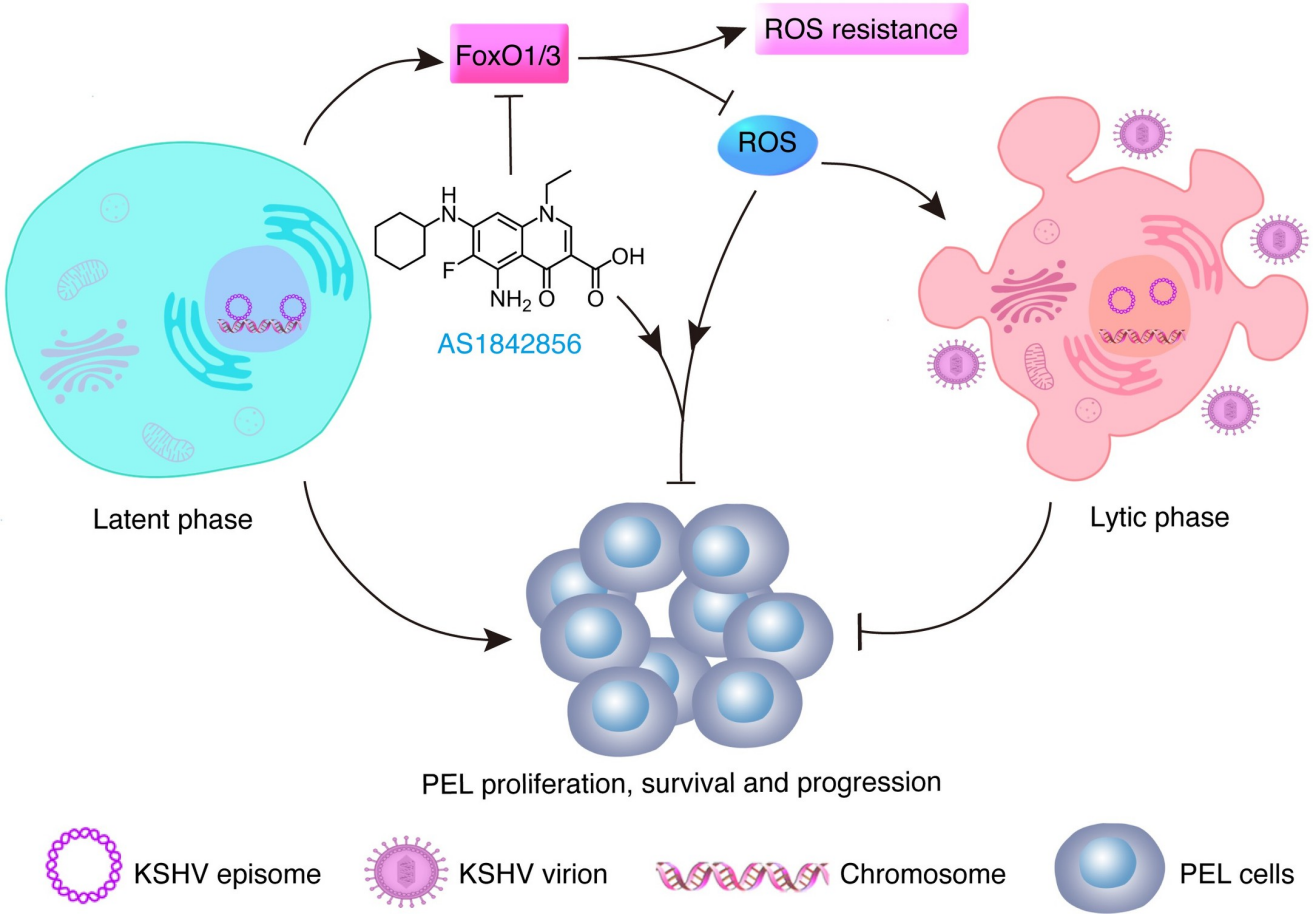
The working model for targeting FoxO proteins for PEL treatment by inducing KSHV lytic reactivation. During KSHV latent infection, FoxO1 was slightly while FoxO3 was robustly upregulated to support PEL proliferation, survival and progression by detoxifying ROS. Upon FoxO inhibition by either shRNAs or AS1842856, the intracellular ROS level was increased, which poses direct cytotoxic effects as well as triggers KSHV lytic reactivation, altogether leading to the massive death of PEL cells.

By perturbing FoxOs expression or functions, the cellular redox balance was disrupted, leading to ROS-dependent viral lytic program and impaired cell viability by inducing apoptosis of PEL cells both *in vitro* and *in vivo*. The induction of KSHV lytic replication achieved by FoxOs inhibition was comparable to NaB that is the well-characterized positive control [54]. The increased ROS caused by FoxOs suppression is speculated to incur two consequences in KSHV-positive B lymphoma cells (Fig 9). On one hand, the accumulated ROS posed the direct cytotoxicity to PEL cells, as FoxOs inhibition disrupts the detoxification system and eventually sensitized PEL cells to H_2_O_2_-induced cell death (S2B-S2F Fig). One the other hand, the elevated level of ROS following FoxOs inhibition, provoked the lytic replication of KSHV, which at least partially accounted for the preferential death of KSHV-positive B lymphoma cells compared to KSHV-negative B lymphoma cells (Figs 4–7). In a xenografted mouse model, we showed that FoxO inhibitor prevented the initiation and progression of PEL *in vivo*. More importantly, the intraperitoneal administration of FoxO inhibitor led to the efficient regression of established PEL (Fig 8F–8H). Further, because of the low solubility of FoxO inhibitor AS1842856 in saline, which potentially limited its effectiveness *in vivo*, a more soluble inhibitor for FoxOs is required for further clinical studies.

Although we have identified FoxO1 and FoxO3 as promising therapeutic targets and AS1842856 as a therapeutic reagent for PEL, we have only examined their effects on BJAB-KSHV and three PEL cell lines so far. It would be necessary to extend our findings to other cell lines by including appropriate controls such as normal B cells and primary lymphoma cells. While antioxidant defense is authenticated to be the downstream effector, other targets of FoxO proteins that contribute to the malignant growth of PEL might also exist. Furthermore, we observed that both FoxO1 and FoxO3 are essential for the proliferation and survival of KSHV-positive B lymphoma cells (Fig 3C–3E and 3A-3C). It might be interesting to exquisitely delineate the role of FoxO1 and FoxO3 by performing genomic knockout and genome-wide sequencing. Further, beside FoxO1, AS1842856 also inhibits FoxO3. Pharmacological inhibitors that specifically target FoxO1 or FoxO3 should be developed to dissect their roles in tumorigenesis.

Altogether, we explored the possibility of viral lytic reactivation as a remedy for virus-associated diseases by exemplifying KSHV latently infected PEL cells. Our findings showed that inhibition of FoxO1 or FoxO3 robustly induces the cytotoxicity by triggering the active lytic replication of KSHV in PEL cells, which is highly dependent on ROS. All these establish FoxO proteins as potential targets for treating PEL.

## Materials and methods

### Ethics statement

All animal studies were performed by complying with the guide for the Use and Care of Laboratory Animals in China. All animal-related experiments were conducted by fulfilling the Animal Use and Care Administration Advisory Committee of the Hunan Normal University (2021–338).

### Cell culture and reagents

BJAB, PEL cells (BCBL1, BC3, and BCP1), rat primary metanephric mesenchymal precursor cells (MM) and KSHV-transformed MM cells (KMM) were obtained from Dr. Qingsong Qin (Shantou University, China) and DG75 and BJAB-KSHV was kindly provided by Dr. Ke Lan (Wuhan University, China). BJAB, BJAB-KSHV and PEL cells were cultured in RPMI1640 (Gibco) supplemented with 10% fetal bovine serum (FBS) (ExCell) and antibiotics (100 μg/mL penicillin and 100 μg/mL streptomycin). MM cells were maintained in Dulbecco’s modified Eagle’s medium (DMEM) (Gibco) supplemented with 10% FBS (ExCell) and antibiotics (100 U/mL penicillin and 100 μg/mL streptomycin). KMM cells were cultured the same as MM cells except adding 200 μg/mL hygromycin. All these cells were maintained at a humidified incubator (Thermo Fisher Scientific) at 37°C with 5% CO_2_.

### Western-blotting analysis

Cells were lysed by 1x laemmeli buffer containing 62.5 mM Tris-HCl, pH 6.8, 2% SDS, 10% glycerol, 2.5% β-mercaptoethano, and 0.01% bromophenol blue, followed by boiling at 95°C for 10 min. Total cell lysates were separated in SDS polyacrylamide gels and then electrophoretically transferred to PVDF membranes that were pretreated with 100% methanol for 5 minutes. The prepared membranes were blocked with 3% skim milk for 1 hour at room temperature and then sequentially incubated with indicated primary antibody overnight at 4°C and secondary antibody for 1 hour at room temperature. The used primary antibodies included rabbit monoclonal antibodies to FoxO1 (Cell Signaling Technology, 2880), FoxO3 (Cell Signaling Technology, 2497), cleaved caspase 3 (Cell Signaling Technology, 9664), β-actin (ABclonal, AC026), and a mouse monoclonal antibody to ORFK8 (Santa Cruz, F33P1). β-actin was used as the loading control.

### Immunofluorescent assay

Treated BCP1 cells (1.5x10^4^) were seeded on polylysine-coated glass coverslips (WHB, 12-CS-LC) for 30 min. Cells on coverslips were fixed with 100% methanol for 30 min at room temperature and then washed with PBST for three times. Fixed cells were then permeabilized with 1% Triton X-100 in PBST for 15 min at room temperature and subsequently blocked with 1% BSA in PBST for 30 min at 37°C. Cells were incubated with a mouse anti-ORFK8 monoclonal antibody (Santa Cruz, F33P1) at a 1:100 dilution at 4°C overnight. The cells were then washed three times with PBST followed by incubation with an Alexa Fluor 488-conjugated goat anti-mouse immunoglobulin G secondary antibody for 60 min at 37°C. The cells were again washed with PBST three times and stained with DAPI (Solarbio, C0060) for 5 min at room temperature, and washed with PBST three times. The coverslips were mounted onto slides using an anti-quenching mounting buffer (Solarbio, S2100). Cells were imaged with a fluorescent microscope (Zeiss, M2).

### Cell proliferation assay

To assess the effect of H_2_O_2_ on cell proliferation, cells seeded at 2x10^5^ per mL were either treated with 0 (H_2_O), 1, 5, 10 μM H_2_O_2_. To examine the effect of AS1842856 (MCE, HY-100596) on cell proliferation, 2x10^5^ cells per mL were treated with 0 (DMSO), 1, 2, and 5 μM AS1842856. To assess the effects of FoxO1 or FoxO3 on cell proliferation, cells at 1.5x10^5^ per mL were infected with lentiviruses harbouring non-targeting shRNA (NT), FoxO1 shRNAs or FoxO3 shRNAs. The number of live cells was counted daily for 3 days after the treatment. Briefly, the suspended cells in culture medium were mixed with 0.4% typan blue solution (Yesen, 40207ES20) in 1:1 ratio. Only the unstained cells that indicated the live cells were counted and calculated by hemacytometer. The total cell number was quantitated by multiplying the defined dilution factor.

### Cell apoptosis assay

Cells seeded at a density of 2x10^5^ per mL were treated with different concentration of H_2_O_2_ or AS1842856 (MCE, HY-100596), or cells at 1.5x10^5^ per mL were untransduced, transduced with NT, FoxO1 shRNAs or FoxO3 shRNAs, for three days. Then, cells were pelleted by centrifuging at 1500 rpm and subsequently washed once with PBS, followed by double staining of PI and phycoerythrin-cyanine 7-conjugated Annexin V (Elabscience, 2GB58JNSER) for 15 min at room temperature. Apoptotic cells were visualized by flow cytometry (Thermo Fisher, Attune NxT), and analyzed by FlowJoV10 (FlowJo, LLC, Ashland, OR). Cells were unstained with both Annexin V and PI indicated live cells; cells were stained with Annexin V but not PI suggested early apoptosis; cells were stained with both Annexin V and PI denoted late apoptosis, and cells were stained with PI but not Annexin V indicated dead cells.

### Lentiviral shRNA knockdown

The pLKO.1 lentiviral vector was used to express NT, FoxO1 and FoxO3 shRNAs. Two complementary sequences of shRNAs oligos were synthesized, annealed, and were ligated into the AgeI and EcoRI sites of linearized pLKO.1 lentiviral vector pLKO.1. The target sequences for FoxO1 were, shRNA-1: GGACATGCTCAGCAGACATCT and shRNA-2: GCATGTTCATTGAGCGCTTAG, and for FoxO3 included shRNA-1: CATGTTCAATGGGAGCTTGGA and shRNA-2: CCACACAGAATGTTGTTGGTT. The lentiviral packaging plasmids (pMD2.G and psPAX2) and lentiviral vectors expressing targeting shRNAs were co-transfected into HEK293T cells by polythylenimine (Polysciences, 23966–2). After 2-day and 3-day transfection, the supernatants of HEK293T cells were harvested and centrifuged at 3000 rpm for 10 min to remove cell debris. DG75, BJAB, BJAB-KSHV and PEL (BC3, BCBL1 and BCP1) cells at 1.5x10^5^ per mL were then subjected to spinning infection at 1500 rpm for 1 h in the presence of 8 μg/mL polybrene. The knockdown efficiency was confirmed by both RT-qPCR and Western blot at day 3 post-transduction.

### Generation of BCBL1-Luc cells

pLenti-CMV-puro-Luc (w168-1) lentiviral plasmid was kindly provided by Dr.Qingsong Qin (Shantou University, China). BCBL1-Luc cells that were generated by lentiviral transduction of the firefly luciferase gene into BCBL1 cells. Briefly, firefly luciferase expression plasmid pLenti was co-transfected with pMD2.G and psPAX2 packaging plasmids into HEK293T cells using polyethyleneimine (Polysciences, 23966–2). At day 3 post-transfection, the supernatants containing lentiviral particles was collected after removing cell debris by centrifuge. BCBL1 cells were then transduced with firefly luciferase (BCBL1-Luc) according to the method mentioned above. BCBL1-Luc cells were selected by 0.5 μg/mL puromycin. Expression of active luciferase protein was confirmed by examining the bioluminescence signal of live BCBL1-Luc cells after incubation with D-luciferin (PerkinElmer, 122799) at a final concentration of 0.5 mg/mL. BCBL1-Luc cells were cultured the same as the PEL cells.

### Flow cytometry detection of ROS

BJAB and PEL cells were treated with AS1842856 at 0 (DMSO), 1, 2, and 5 μM for 48 h; BCBL1 or BCP1 cells untreated or transduced with non-targeting (NT) shRNA and FoxO1 or FoxO3 shRNAs for 72 h were subjected to flow cytometry for ROS detection. Briefly, treated cells were loaded with ROS dye, that is, 5-(and -6)-carboxy-2,7-dichlorodihy drofluorescein diacetate (H2DCFDA) (Biosharp, BL714A), at a final concentration of 10 μM for 1 hour at 37°C. Cells were then washed once with PBS to remove unbound ROS dye and then subjected to flow cytometry analysis. To examine the effects of N-acetyl cysteine (NAC) on ROS, cells were pretreated with 400 μM NAC daily for two days before adding ROS dye.

### RNA isolation and RT-qPCR

Cells were pelleted and lysed by TRIzol Reagent (TransGen Biotech, ET111-01). Total RNA was isolated by chloroform extraction and followed by isopropanol precipitation. 1 μg total RNA was used for reverse transcription following the instructions of the cDNA synthesis kit (TransGen Biotech, P40719). qPCR analysis of cDNA was done using SYBR Green (TransGen Biotech, Q20310) and all qPCR reaction were run in triplicate. The relative gene expression was normalized with the internal control β-actin, yielding 2^-ΔΔCt^ values. The primers used for qPCR are 5′- TTGCCGACAGGATGCAGAAG-3′ (F) and 5′-GTACTTGCGCTCAGGAGGAG-3′ (R) for β-actin; 5′-AAGAGCGTGCCCTACTTCAA-3′ (F) and 5′-CTGTTGTTGTCCATGGATGC-3′ (R) for FoxO1; 5′-GCAGACACTGAAACGCTGAA-3′ (F) and 5′-AGGTGAGCCACCAGGACTTA-3′ (R) for LANA; 5′-CACAAAAATGGCGCAAGATGA-3′ (F) and 5′-TGGTAGAGTTGGGCCTTCAGTT-3′ (R) for RTA; 5′-TTTAGCACTGGGACTGCCC-3′ (F) and 5′-CAAGAAGGCAAGCAGCGAG-3′ (R) for PAN RNA; 5′-CATGCTGATGCGAATGTGC-3′ (F) and 5′-AGCTTCAACATGGTGGGAGTG-3′ (R) for ORFK8; 5′-AGGTCCCCCTCACCAGTAAA-3′ (F) and 5′-GAGGACGTGTGTTTTGACCG-3′ (R) for ORF57; 5′-CGAGTCTTCGCAAAAGGTTC-3′ (F) and 5′-AAGGGACCAACTGGTGTGAG-3′ (R) for ORF59; 5′-ATATGTCGCAGGCCGAATA-3′ (F) and 5′-CCACCCATCCTCCTCAGATA-3′ (R) for ORF65.

### Measurement of virion production

Supernatants from treated cells were collected and cleared by centrifugation at 1000 g for 3 min. Cleared supernatants were then treated with 100 U/mL DNase I (Invitrogen, P30727) for 30 min at 37°C. DNase I was subsequently inactivated by heating at 65°C for 30 min with 10 mM EDTA. The number of KSHV episomal DNA copies were calculated by quantitative PCR using primers specific for KSHV ORF65 (sense: 5’- ATATGTCGCAGGCCGAATA-3’, anti-sense: 5’- CCACCCATCCTCCTCAGATA -3’). The pCDH plasmid carrying KSHV ORF65 cDNA was used to generate the standard curve.

### Animal experiments

NOD/SCID mice, all at 6 weeks old, were kept under pathogen-free conditions. To establish the PEL tumor model, 1x10^7^ BCBL1-Luc cells in 200 μL DMEM were intraperitoneally (i.p.) injected per NOD/SCID mouse.

For the tumor initiation experiment, 14 NOD/SCID mice were prepared and each of them was i.p. injected with 1x10^7^ BCBL1-Luc cells. At day 1 post-inoculation, mice were i.p. injected with 15 mg/kg AS1842856 (Adooq Bioscience, A15871) or vehicle (6% 2-hydroxypropyl-β-cyclodextrin and 5% DMSO in saline) (MCE, HY-101103) twice per day for consecutive 2 weeks. Mice were weighed every two days in the morning. On day 7 and day 14 post inoculation, mice were subjected to live imaging, starting with i.p. injection of D-luciferin (PerkinElmer, 122799) at a final concentration of 50 mg/kg, followed by 10 minutes incubation time. Then mice were imaged with the IVIS Lumina LT system for 10 second.

For the tumor regression experiment, 8 NOD/SCID mice i.p. injected with 1x10^7^ BCBL1-Luc cells for 16 days were randomly divided into two subgroups. Then, the mice were treated with either 15 mg/kg AS1842856 (Adooq Bioscience, A15871) or vehicle (6% 2-hydroxypropyl-β-cyclodextrin and 5% DMSO in saline) two times per day for 4 days, followed by three times per day for consecutive 1 weeks. The body weight of mice was measured every two days in the morning. Mice were imaged at day 7 and day 14 post treatment of AS1842856 (i.e., 23 days and 27 days post inoculation). Mice were euthanized to harvest ascites either because of the abnormal gait or impaired health or at day 27 post inoculation. The region-of-interest (ROI) signals based on the (p/s)/ (microwatts/square centimeter) formula were analyzed with Living Image software (IVIS Imaging System).

### Statistical analysis

Data were present as the mean ± standard error of the mean (SEM) from at least three independent experiments except specified. Statistical analysis was performed using the two-tailed t-test between two groups, and one-way ANOVA was applied to analyze the differences more than two groups except specifically stated. A *P* value of ≤0.05 was considered statistically significant. *, ** and *** mean *P*-values < 0.05, < 0.01 and < 0.001, respectively, while “ns” indicates “not significant”.

## Supporting information

S1 Fig FoxO1 or FoxO3 is knockded down by specific shRNAs. (A-B) The mRNA and protein expression of FoxO1 were examined by RT-qPCR (A) and Western blot (B) in BJAB and PEL (BCP1, BC3, and BCBL1) cells infected with lentiviruses harbouring non-targeting (NT) or FoxO1 shRNAs (sh1 or sh2) for 3 days. (C) The protein level of FoxO3 in BJAB and PEL cells transduced with NT shRNAs or FoxO3 shRNAs (sh1 or sh2) for 3 days was analyzed by Western blot. *, *P* < 0.05, **, *P* <0.01, ***, *P* < 0.001, ns, not significant compared to NT.
(TIF)

S2 Fig Inhibition of FoxO1 suppresses the proliferation and induces apoptosis of PEL cells. (A) The protein expression of FoxO1 and FoxO3 in PEL cells was examined by Western blot. (B-C) Flow cytometry detection of the intracellular ROS level using c-H2DCFDA staining in BC3 cells treated with 1 μM AS1842856 for 72 h, followed by the treatment of indicated concentrations of H_2_O_2_ for 1 h. Representative images were shown in (B), and the quantification for B from three independent repeats, were presented in (C). (D) The cell viability of BJAB and BJAB-KSHV cells following the treatment of different dosages of AS1842856 for 72 h was indicated, and the IC_50_ of AS1842856 was calculated. (E) The proliferation curve of BC3 cells treated with 1 μM AS1842856, indicated concentrations of H_2_O_2_, or both for 24, 48, and 72 h. (F) Flow cytometry analysis of apoptosis in BC3 cells treated with 1 μM AS1842856, indicated concentrations of H_2_O_2_ or both for 72 h. BC3 cells unstained with both Annexin V and PI indicated live cells; cells only stained with Annexin V indicated early apoptosis; cells stained with both Annexin V and PI indicated late apoptosis and cells only stained with PI indicated dead cells. All the values were shown as mean ± SEM. *, *P* < 0.05, **, *P* <0.01, ***, *P* < 0.001, ns, not significant.
(TIF)

S3 Fig FoxO3 knockdown inhibits the proliferation and induces the apoptosis of KSHV-positive B lymphoma cells. (A) FoxO3 and c-caspase 3 protein expression in BJAB, BJAB-KSHV and PEL cells infected with lentiviruses harbouring NT or FoxO3 shRNAs (sh1 or sh2) for 3 days were examined by Western blot. (B) The proliferation curves of BJAB, BJAB-KSHV and PEL cells untransduced, transduced with NT or FoxO3 shRNAs for 1 day, 2 days, and 3 days. (C) Flow cytometry detection of apoptosis using Annexin V and PI staining in BJAB, BJAB-KSHV and PEL cells after 72 h post-transduction of NT or FoxO3 shRNAs. (D) Western blotting analysis of the protein expression of FoxO1, FoxO3 and c-caspase 3 in BC3 and BCBL1 cells after 72 h post-transduction of NT shRNA, FoxO1 shRNA-sh1, FoxO3 shRNA-sh1 or both. (E) The proliferation curves of BC3 and BCBL1 cells untransduced, transduced with NT shRNA, FoxO1 shRNA-sh1, FoxO3 shRNA-sh1 or both for 1 day, 2 days, and 3 days. (F). Flow cytometry detection of apoptosis using Annexin V and PI staining in BC3 and BCBL1 cells transduced with NT shRNA, FoxO1 shRNA-sh1, FoxO3 shRNA-sh1 or both for 72 h. Cells unstained with both Annexin V and PI indicated live cells; cells only stained with Annexin V indicated early apoptosis; cells stained with both Annexin V and PI indicated late apoptosis and cells only stained with PI indicated dead cells. **, *P* <0.01, ***, *P* < 0.001, ns, not significant compared to NT or as indicated.
(TIF)

S4 Fig FoxO inhibitor AS1842856 induces KSHV lytic replication. (A-B) RT-qPCR analysis of the mRNA levels of KSHV LANA, RTA, PAN RNA, ORFK8, ORF57, ORF59 and ORF65 in BC3 (A) and BCBL1 (B) cells treated with 0, 1, 2, 5 μM AS1842856 or 0.3 mM sodium butyrate (NaB) for 72 h. (C-D) The quantitation of the produced KSHV virion in the supernatant of BC3 (C) and BCBL1 (D) cells treated with 0, 1, 2, 5 μM AS1842856 or 0.3 mM NaB for 96 h by qPCR. *, *P* < 0.05, **, *P* <0.01, ***, *P* < 0.001, ns, not significant compared to 0 μM AS1842856.
(TIF)

S5 Fig FoxO1 and FoxO3 knockdown triggers KSHV lytic replication. (A-B) RT-qPCR analysis of the mRNA levels of KSHV LANA, RTA, PAN RNA, ORFK8, ORF57, ORF59 and ORF65 in BC3 (A) and BCBL1 (B) cells after 72 h post-transduction of NT shRNA, FoxO1 shRNAs or FoxO3 shRNAs. *, *P* < 0.05, **, *P* <0.01, ***, *P* < 0.001, ns, not significant by 2-tailed Student’s t test compared to NT.
(TIF)

S6 Fig FoxO1 inhibition triggers KSHV lytic reactivation in a ROS-dependent manner. (A-B) RT-qPCR detection of the transcript expression of KSHV lytic genes including PAN RNA, RTA, ORFK8, ORF65, ORF57, and ORF59 in BC3 (A) and BCBL1 (B) cells treated with 0 or 5 μM AS1842856 with or without the daily treatment of 0.4 mM for 3 days. ***, *P* < 0.001, ns, not significant.
(TIF)

S7 Fig AS1842856 inhibits PEL tumor initiation, progression and regression *in vivo* by inducing KSHV reactivation. (A) Bioluminescence imaging of BCBL1-Luc at indicated cell numbers in a 96-well plate. (B) The number of BCBL1-Luc cells was linearly correlated to the total bioluminescence flux. (C) Photograph of organs including lung, heart, spleen, liver, pancreas, and kidney of mice treated with AS1842856. (D) Photograph of solid tumors in the hypodermis of mice treated with vehicle or AS1842856. (E) Photograph of organs including lung, heart, spleen, liver, pancreas, and kidney of mice treated with vehicle or AS1842856. (F) RT-qPCR analysis of the mRNA levels of KSHV lytic genes including RTA, ORFK8, ORF59, ORF65 in ascites from mice treated with vehicle or AS1842856.
(TIF)
