# Supplementary figures and images for: Targeting FoxO proteins induces lytic reactivation of KSHV for treating herpesviral primary effusion lymphoma
Source: PLoS Pathog. 2023 Aug 18;19(8):e1011581. doi: 10.1371/journal.ppat.1011581 (PMC10468091; doi:10.1371/journal.ppat.1011581)

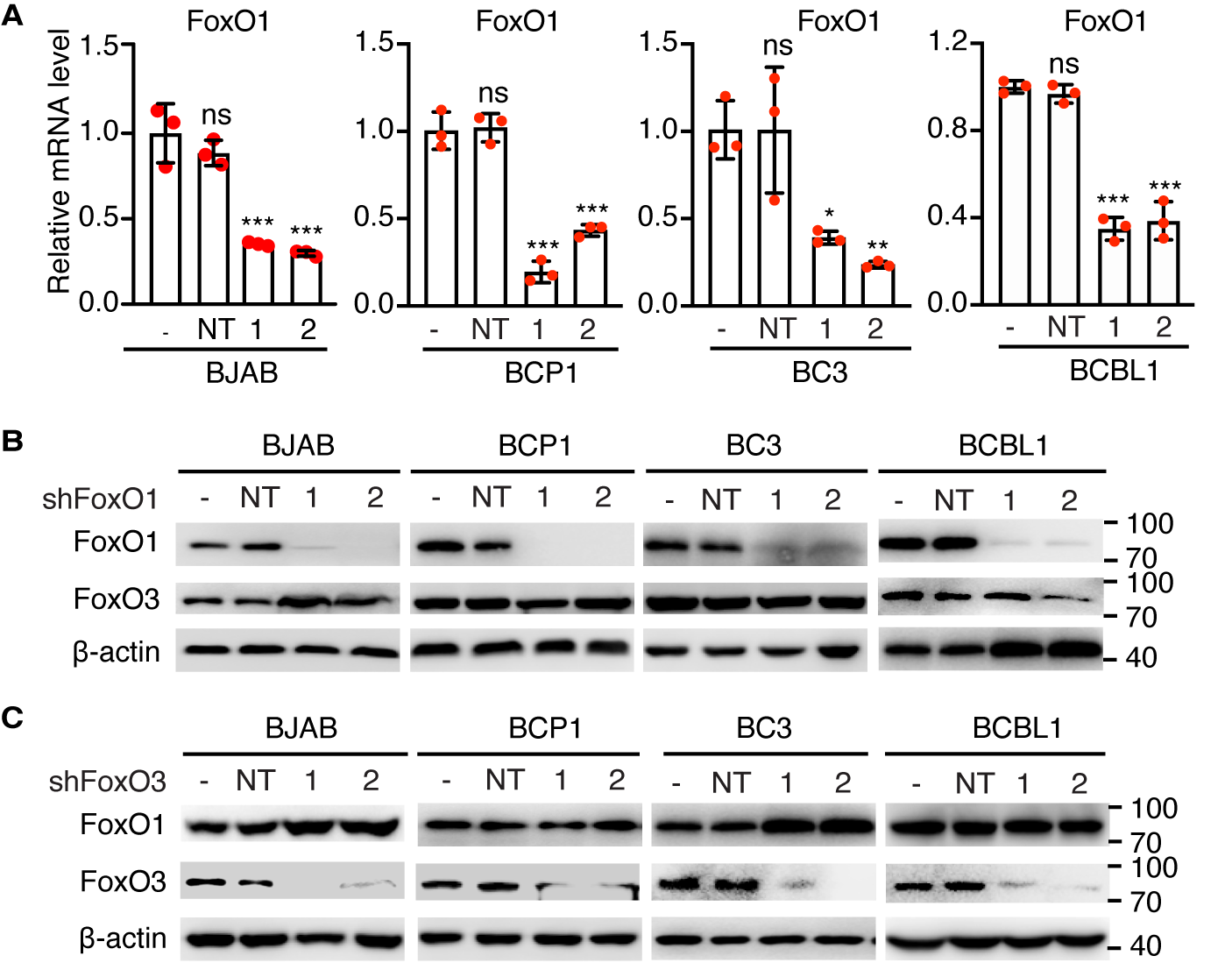

Supplement: S1 Fig — (A-B) The mRNA and protein expression of FoxO1 were examined by RT-qPCR (A) and Western blot (B) in BJAB and PEL (BCP1, BC3, and BCBL1) cells infected with lentiviruses harbouring non-targeting (NT) or FoxO1 shRNAs (sh1 or sh2) for 3 days. (C) The protein level of FoxO3 in BJAB and PEL cells transduced with NT shRNAs or FoxO3 shRNAs (sh1 or sh2) for 3 days was analyzed by Western blot. *, P < 0.05, **, P <0.01, ***, P < 0.001, ns, not significant compared to NT. (TIF) [file ppat.1011581.s001.tif]

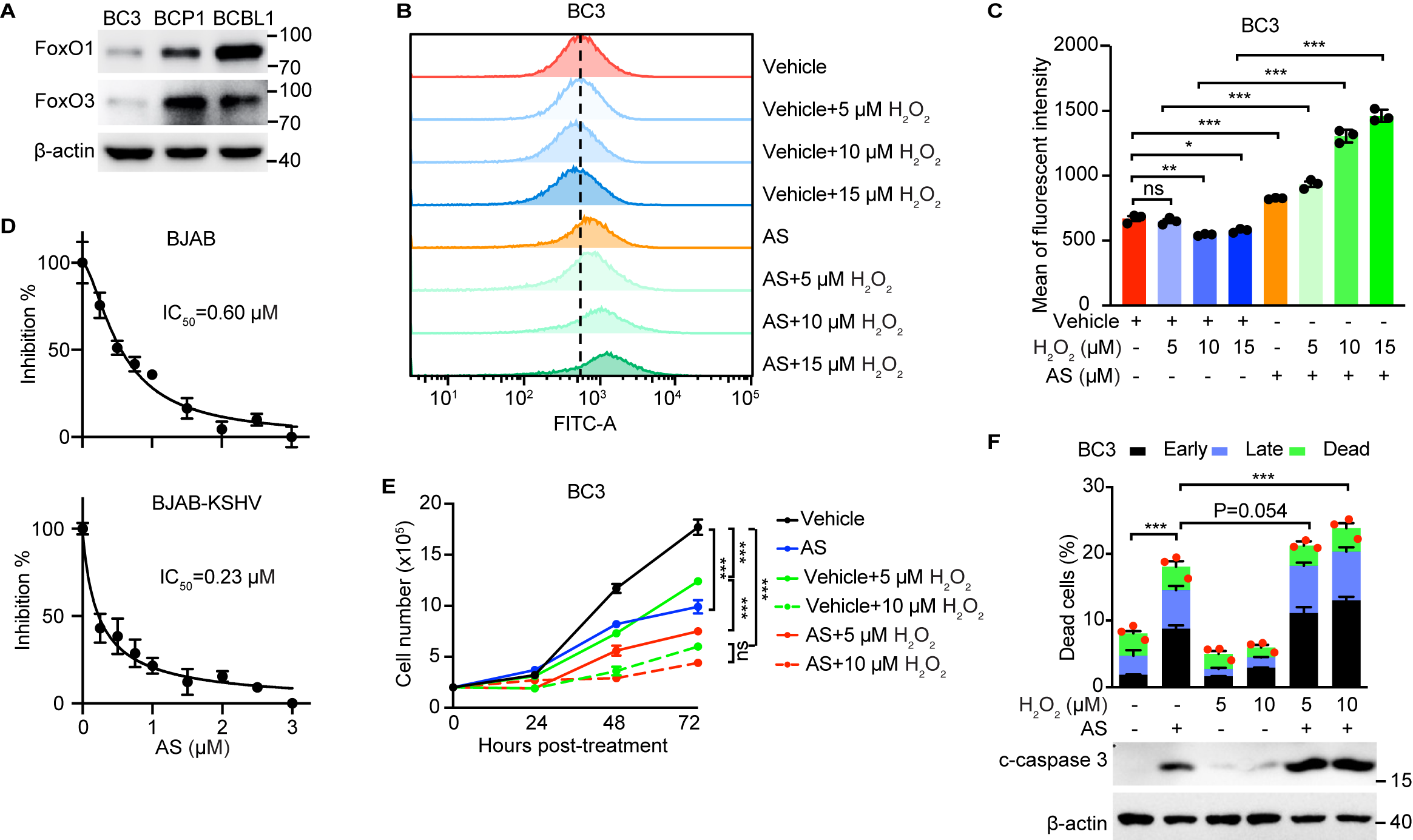

Supplement: S2 Fig — (A) The protein expression of FoxO1 and FoxO3 in PEL cells was examined by Western blot. (B-C) Flow cytometry detection of the intracellular ROS level using c-H2DCFDA staining in BC3 cells treated with 1 μM AS1842856 for 72 h, followed by the treatment of indicated concentrations of H2O2 for 1 h. Representative images were shown in (B), and the quantification for B from three independent repeats, were presented in (C). (D) The cell viability of BJAB and BJAB-KSHV cells following the treatment of different dosages of AS1842856 for 72 h was indicated, and the IC50 of AS1842856 was calculated. (E) The proliferation curve of BC3 cells treated with 1 μM AS1842856, indicated concentrations of H2O2, or both for 24, 48, and 72 h. (F) Flow cytometry analysis of apoptosis in BC3 cells treated with 1 μM AS1842856, indicated concentrations of H2O2 or both for 72 h. BC3 cells unstained with both Annexin V and PI indicated live cells; cells only stained with Annexin V indicated early apoptosis; cells stained with both Annexin V and PI indicated late apoptosis and cells only stained with PI indicated dead cells. All the values were shown as mean ± SEM. *, P < 0.05, **, P <0.01, ***, P < 0.001, ns, not significant. (TIF) [file ppat.1011581.s002.tif]

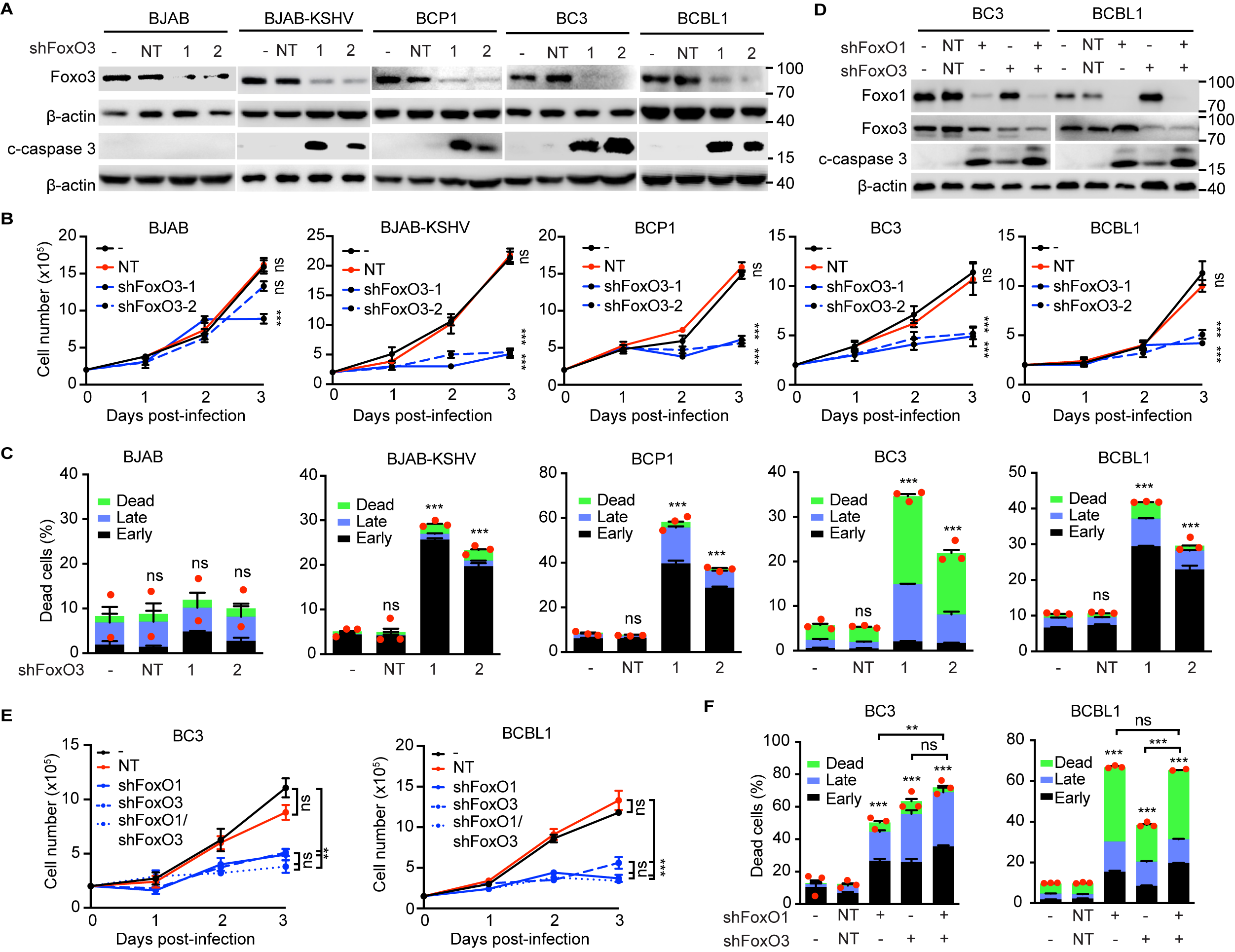

Supplement: S3 Fig — (A) FoxO3 and c-caspase 3 protein expression in BJAB, BJAB-KSHV and PEL cells infected with lentiviruses harbouring NT or FoxO3 shRNAs (sh1 or sh2) for 3 days were examined by Western blot. (B) The proliferation curves of BJAB, BJAB-KSHV and PEL cells untransduced, transduced with NT or FoxO3 shRNAs for 1 day, 2 days, and 3 days. (C) Flow cytometry detection of apoptosis using Annexin V and PI staining in BJAB, BJAB-KSHV and PEL cells after 72 h post-transduction of NT or FoxO3 shRNAs. (D) Western blotting analysis of the protein expression of FoxO1, FoxO3 and c-caspase 3 in BC3 and BCBL1 cells after 72 h post-transduction of NT shRNA, FoxO1 shRNA-sh1, FoxO3 shRNA-sh1 or both. (E) The proliferation curves of BC3 and BCBL1 cells untransduced, transduced with NT shRNA, FoxO1 shRNA-sh1, FoxO3 shRNA-sh1 or both for 1 day, 2 days, and 3 days. (F). Flow cytometry detection of apoptosis using Annexin V and PI staining in BC3 and BCBL1 cells transduced with NT shRNA, FoxO1 shRNA-sh1, FoxO3 shRNA-sh1 or both for 72 h. Cells unstained with both Annexin V and PI indicated live cells; cells only stained with Annexin V indicated early apoptosis; cells stained with both Annexin V and PI indicated late apoptosis and cells only stained with PI indicated dead cells. **, P <0.01, ***, P < 0.001, ns, not significant compared to NT or as indicated. (TIF) [file ppat.1011581.s003.tif]

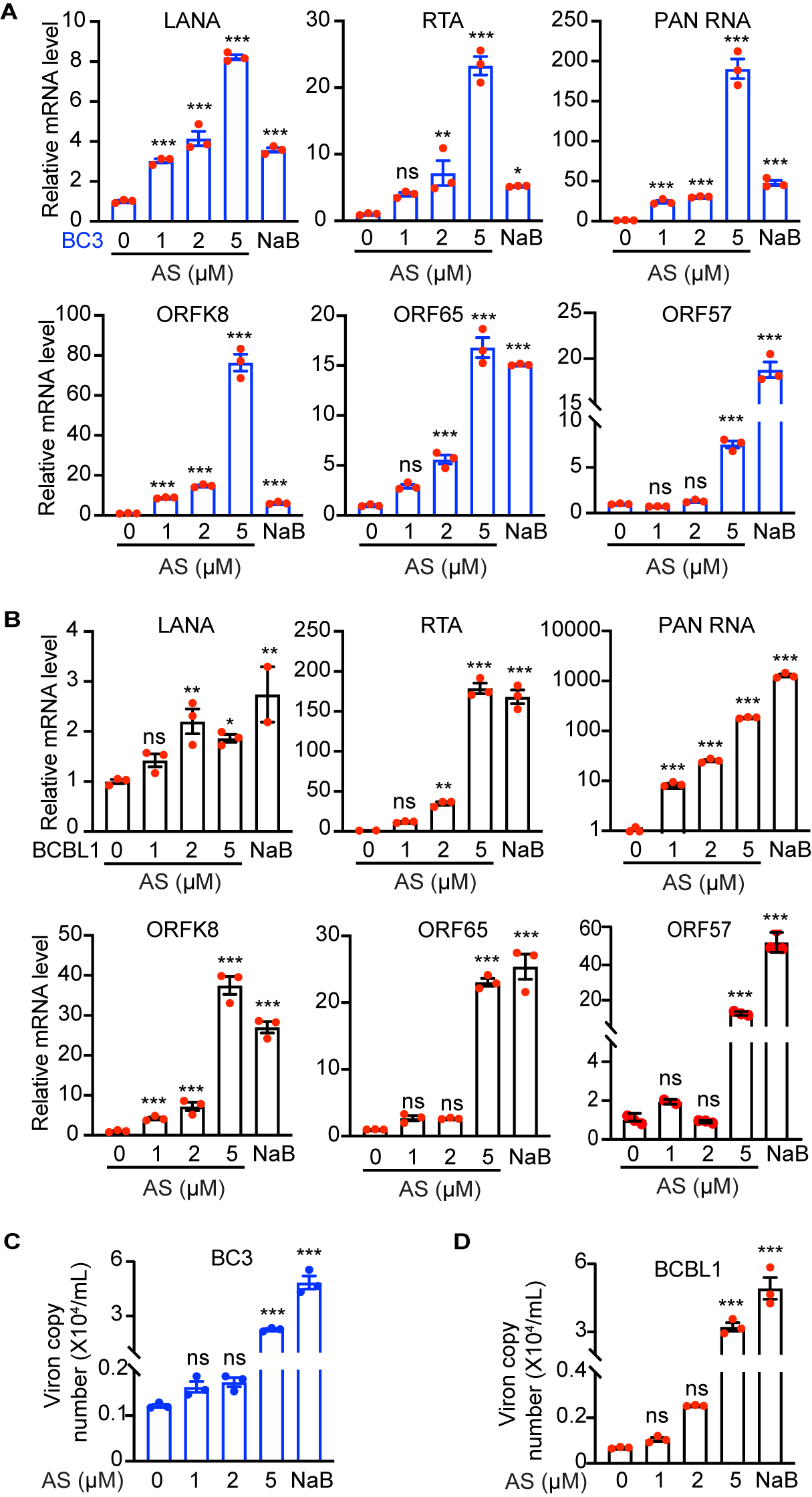

Supplement: S4 Fig — (A-B) RT-qPCR analysis of the mRNA levels of KSHV LANA, RTA, PAN RNA, ORFK8, ORF57, ORF59 and ORF65 in BC3 (A) and BCBL1 (B) cells treated with 0, 1, 2, 5 μM AS1842856 or 0.3 mM sodium butyrate (NaB) for 72 h. (C-D) The quantitation of the produced KSHV virion in the supernatant of BC3 (C) and BCBL1 (D) cells treated with 0, 1, 2, 5 μM AS1842856 or 0.3 mM NaB for 96 h by qPCR. *, P < 0.05, **, P <0.01, ***, P < 0.001, ns, not significant compared to 0 μM AS1842856. (TIF) [file ppat.1011581.s004.tif]

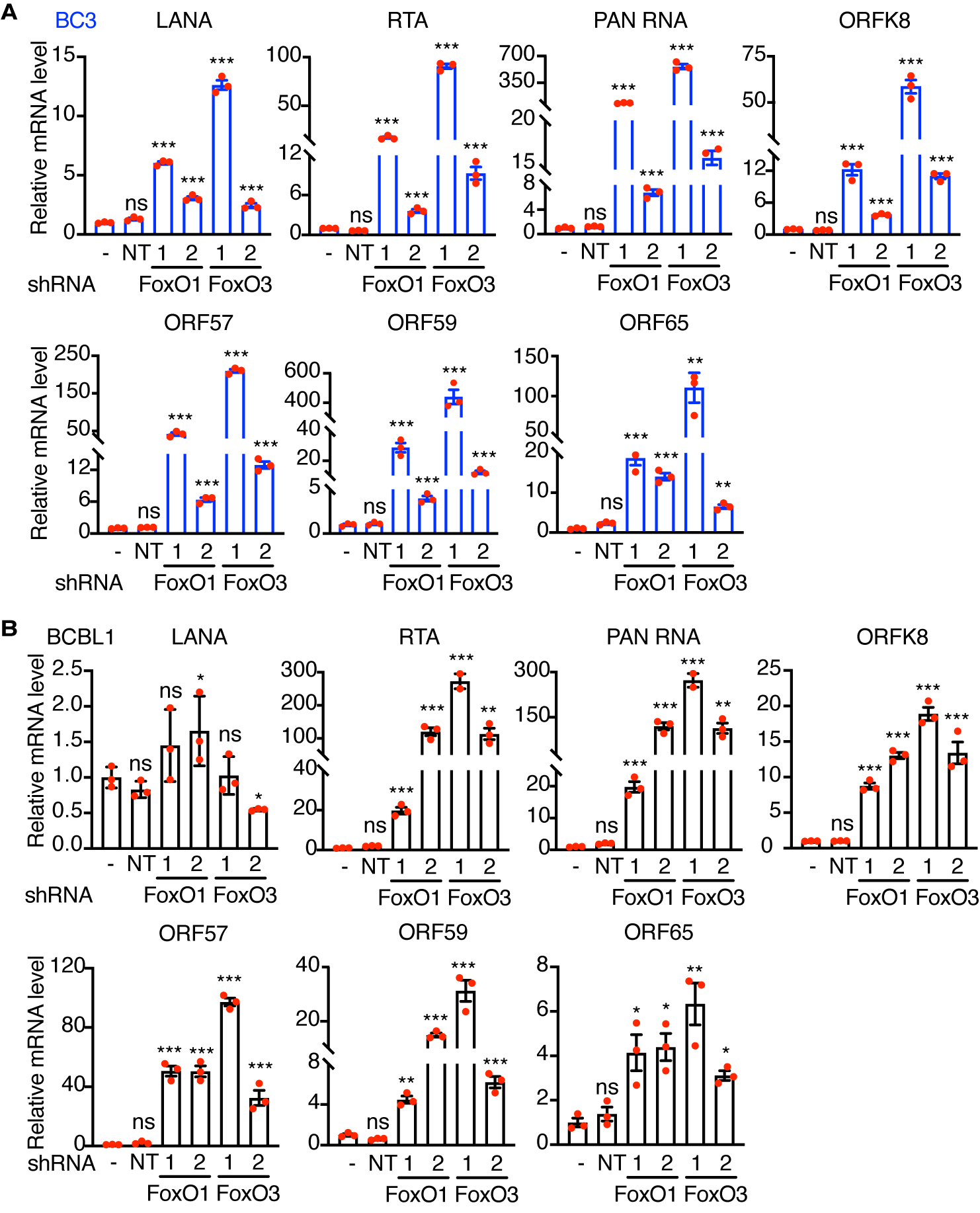

Supplement: S5 Fig — (A-B) RT-qPCR analysis of the mRNA levels of KSHV LANA, RTA, PAN RNA, ORFK8, ORF57, ORF59 and ORF65 in BC3 (A) and BCBL1 (B) cells after 72 h post-transduction of NT shRNA, FoxO1 shRNAs or FoxO3 shRNAs. *, P < 0.05, **, P <0.01, ***, P < 0.001, ns, not significant by 2-tailed Student’s t test compared to NT. (TIF) [file ppat.1011581.s005.tif]

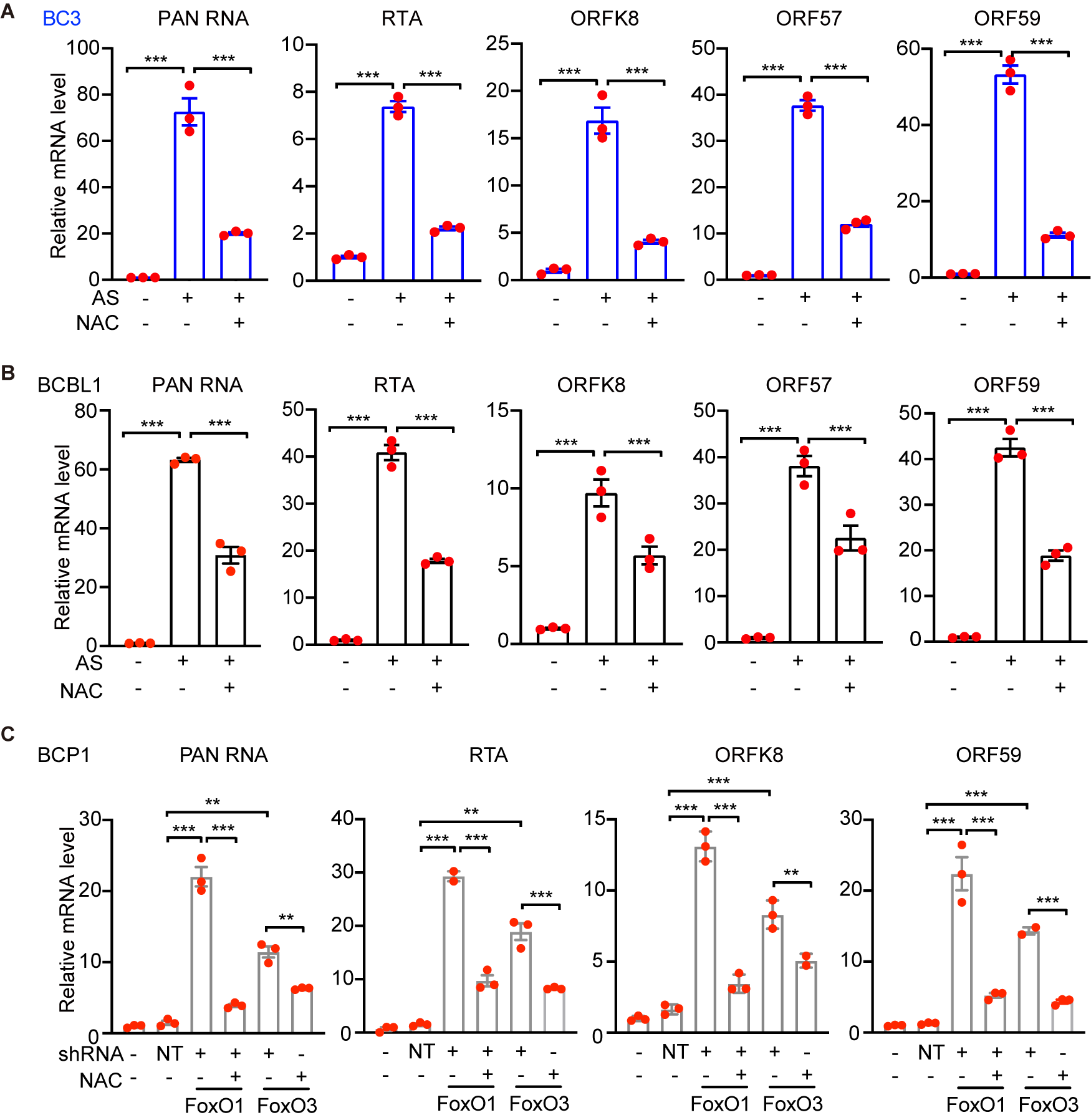

Supplement: S6 Fig — (A-B) RT-qPCR detection of the transcript expression of KSHV lytic genes including PAN RNA, RTA, ORFK8, ORF65, ORF57, and ORF59 in BC3 (A) and BCBL1 (B) cells treated with 0 or 5 μM AS1842856 with or without the daily treatment of 0.4 mM for 3 days. ***, P < 0.001, ns, not significant. (TIF) [file ppat.1011581.s006.tif]

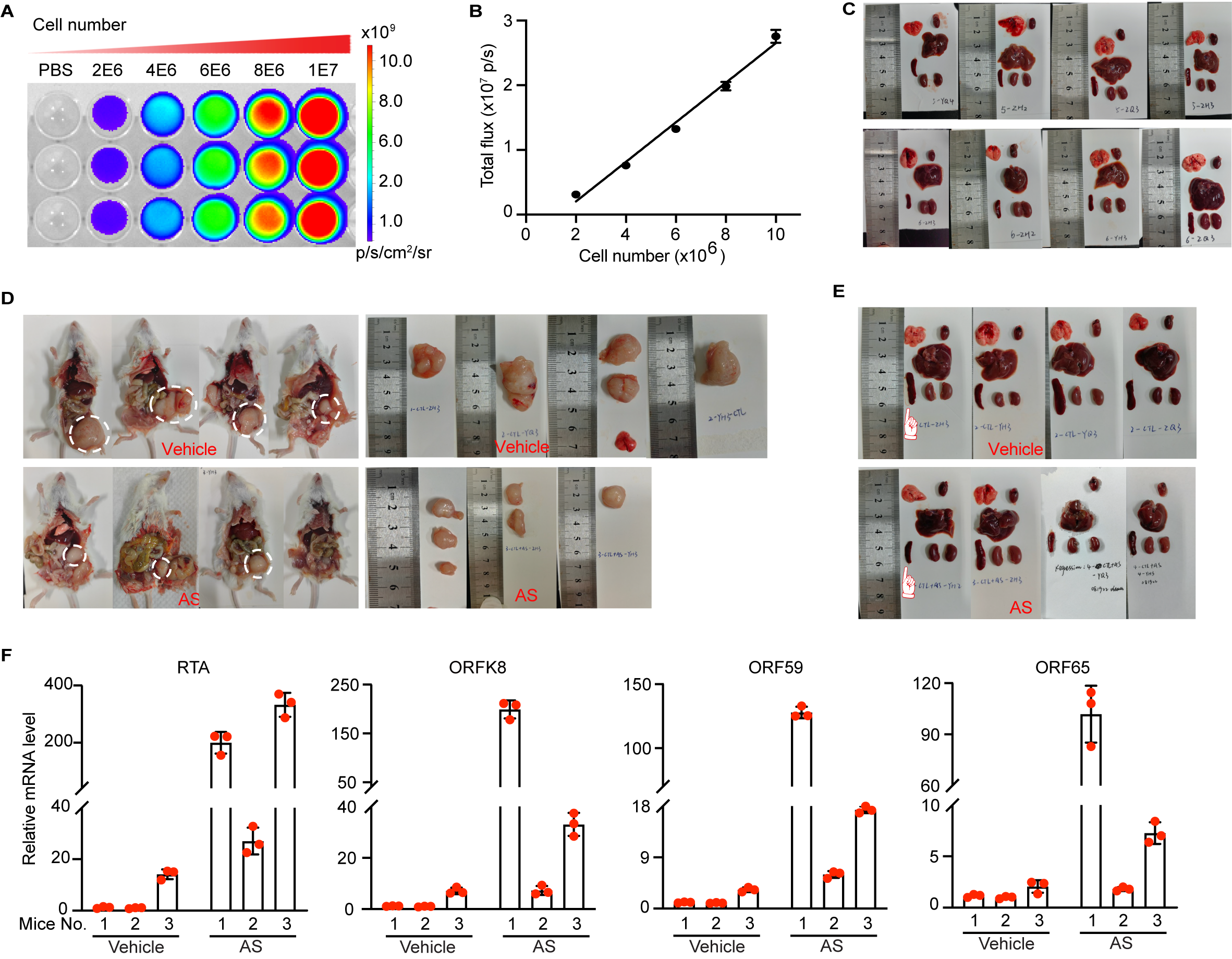

Supplement: S7 Fig — (A) Bioluminescence imaging of BCBL1-Luc at indicated cell numbers in a 96-well plate. (B) The number of BCBL1-Luc cells was linearly correlated to the total bioluminescence flux. (C) Photograph of organs including lung, heart, spleen, liver, pancreas, and kidney of mice treated with AS1842856. (D) Photograph of solid tumors in the hypodermis of mice treated with vehicle or AS1842856. (E) Photograph of organs including lung, heart, spleen, liver, pancreas, and kidney of mice treated with vehicle or AS1842856. (F) RT-qPCR analysis of the mRNA levels of KSHV lytic genes including RTA, ORFK8, ORF59, ORF65 in ascites from mice treated with vehicle or AS1842856. (TIF) [file ppat.1011581.s007.tif]
